# Single-atom dispersed Co–N–C catalyst: structure identification and performance for hydrogenative coupling of nitroarenes†
†Electronic supplementary information (ESI) available: Experimental details, catalyst characterization and tests. See DOI: 10.1039/c6sc02105k


**DOI:** 10.1039/c6sc02105k

**Published:** 2016-06-13

**Authors:** Wengang Liu, Leilei Zhang, Wensheng Yan, Xiaoyan Liu, Xiaofeng Yang, Shu Miao, Wentao Wang, Aiqin Wang, Tao Zhang

**Affiliations:** a State Key Laboratory of Catalysis , iChEM (Collaborative Innovation Center of Chemistry for Energy Materials) , Dalian Institute of Chemical Physics , Chinese Academy of Sciences , 457 ZhongShan Road , Dalian , 116023 , China . Email: aqwang@dicp.ac.cn ; Email: taozhang@dicp.ac.cn; b University of Chinese Academy of Sciences , Beijing , 100049 , China; c National Synchrotron Radiation Laboratory , University of Science and Technology of China , Hefei , 230029 , China

## Abstract

Co–N–C catalysts are promising candidates for substituting platinum in electrocatalysis and organic transformations. The heterogeneity of the Co species resulting from high-temperature pyrolysis, however, encumbers the structural identification of active sites. Herein, we report a self-supporting Co–N–C catalyst wherein cobalt is dispersed exclusively as single atoms. By using sub-Ångström-resolution HAADF-STEM in combination with XAFS and DFT calculation, the exact structure of the Co–N–C is identified to be CoN_4_C_8_-1-2O_2_, where the Co center atom is coordinated with four pyridinic N atoms in the graphitic layer, while two oxygen molecules are weakly adsorbed on Co atoms in perpendicular to the Co–N_4_ plane. This single-atom dispersed Co–N–C catalyst presents excellent performance for the chemoselective hydrogenation of nitroarenes to produce azo compounds under mild reaction conditions.

## Abstract 2

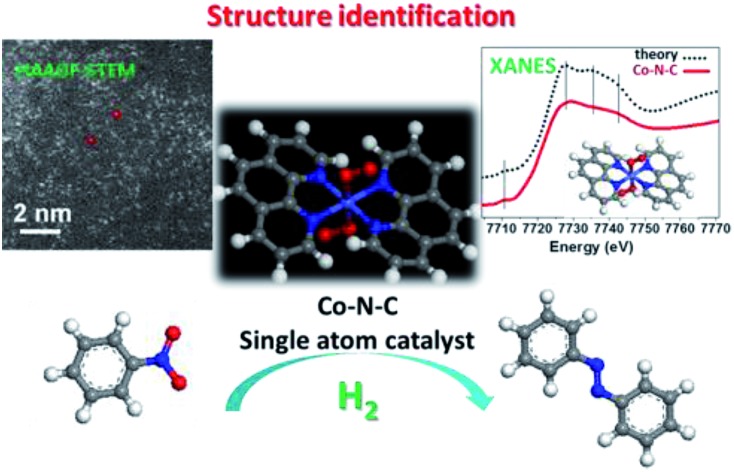
The single-atom Co–N–C catalyst with the structure of CoN_4_C_8_-1-2O_2_ shows excellent performance for the chemoselective hydrogenation of nitroarenes to produce azo compounds under mild reaction conditions.

## Introduction

M–N–C (M refers to transition metals, typically Fe and Co), a type of metal and nitrogen bi-modified carbon material which is usually evolved from the pyrolysis of metal complexes with macrocyclic N_4_ ligands, has been widely investigated in electrocatalysis as a potential substitute for platinum.^1^–^7^ Owing to their capability of activating both hydrogen and oxygen, this class of noble-metal-free catalysts has recently been explored for a variety of hydrogenation and oxidation reactions in organic synthesis,^8^–^13^ such as reduction of nitroarenes to anilines,^9^,^10^ esterification of alcohols,^11^,^12^ and oxidation of ethylbenzene.^13^ One key issue associated with the catalysis of Co(Fe)–N–C materials is the identification of active sites.^3^,^14^–^19^ As most of the catalysts prepared by pyrolysis at high temperatures (600–900 °C) are composed of nanoparticles ranging from a few to tens of nanometers, both exposed and encapsulated, as well as of dispersed single atoms that are invisible with normal electron microscopy techniques, the unambiguous identification of active sites is still a pending challenge. In literature reports, big CoO_*x*_ nanoparticles,^3^ encapsulated Fe or FeO_*x*_ nanoparticles,^15^,^20^ as well as invisible FeN_*x*_ were all respectively assumed as the active sites,^4^,^16^ without compelling evidence. Very recently, we prepared a Co–N–C catalyst on mesoporous carbon and investigated its catalytic capability for aerobic oxidative cross-coupling of primary and secondary alcohols.^8^ By conducting control experiments of acid leaching and extensive characterization of the catalysts before and after acid treatment, we proposed that the single Co atoms bonded with N within graphitic layers were catalytically active sites, whereas those particles of Co or CoO_*x*_ were merely spectators. Nevertheless, this claim is still to be proved unequivocally given that multiple species of cobalt co-exist in the catalyst. On the other hand, the heterogeneity of such catalysts, not only complicates the understanding of the catalytic mechanism, but also greatly decreases the atomic efficiency, and even results in undesirable side-reactions. To tackle these problems, it is highly desirable to synthesize a single-atom M–N–C catalyst wherein M is exclusively dispersed as single atoms by bonding with N atoms.

In fact, single-atom catalysts (SACs) are emerging as a new frontier in heterogeneous catalysis;^21^–^25^ in particular, various supported noble metal SACs have shown great potential in a plethora of chemoselective hydrogenation reactions attributed to the uniform and isolated active sites, *e.g.*, chemoselective hydrogenation of nitroarenes to anilines,^24^ selective hydrogenation of acetylene to ethylene,^26^ and 1,3-butadiene to 1-butylene.^27^,^28^ However, there is no report on the successful application of noble-metal-free SACs to any chemoselective hydrogenation reactions due to the difficulty in preparing such SACs. Very recently, a report on Co SAC supported on N-doped graphene was published, which exhibited superior activity as an electrocatalyst for the hydrogen evolution reaction (HER).^29^ Unfortunately, a clear image of the exact structure of the active site is still lacking.

Herein, we report the successful synthesis of a self-supporting Co–N–C catalyst with single-atom dispersion by using a support-sacrificed approach. The unrivalled uniformity of Co species provides a good entry to the identification of active sites without ambiguity. By using sub-Ångström-resolution high-angle annular dark field aberration-corrected scanning transmission electron microscopy (HAADF-STEM), X-ray absorption spectroscopy (XAS), and density functional theory (DFT) calculation, we provide compelling evidence that the exact structure of the active site is CoN_4_C_8_-1-2O_2_ where Co center atom is coordinated with four pyridinic N atoms in the graphitic layer while two oxygen molecules are weakly adsorbed on Co atoms perpendicular to the Co–N_4_ plane. To the best of our knowledge, this is the first report on the identification of Co–N–C structure. This single-atom Co–N–C catalyst exhibited excellent activity and selectivity for the hydrogenative coupling of nitroarenes to synthesize aromatic azo compounds. A variety of reducible functionalities were well tolerated in the catalytic system, and the corresponding azo products could be obtained with good to excellent yields.

## Results and discussion

### Preparation of the self-supporting Co–N–C catalyst

The self-supporting Co–N–C catalyst with single-atom dispersion was prepared by using a support-sacrificial approach. Briefly, a Co(phen)_2_(OAc)_2_ complex precursor was first supported on Mg(OH)_2_ and then the mixture was submitted to pyrolysis at 700 °C in N_2_ for 2 h, after which the MgO support was removed by treating the material with nitric acid. Elemental analysis of the as-prepared Co–N–C catalyst revealed a Co loading of 3.6 wt% while the Mg residue was almost negligible (0.09 wt%). In comparison with the commonly used carbon supports, the employment of Mg(OH)_2_ presented the prominent advantages of preventing the aggregation of cobalt (Fig. S1†), which could be attributed to the moderate interaction of Mg(OH)_2_ with the Co species as well as its inertness towards the reaction with Co during high temperature pyrolysis process; both of these facilitated the dispersion of the Co species. Furthermore, the support material could be easily removed by acid leaching, which resulted in a self-supporting Co–N–C material. The nitrogen sorption isotherms of the Co–N–C catalyst showed a typical type IV isotherm with a hysteresis loop, indicating a mesoporous structure (Fig. S2†). The BET surface area was 679.9 m^2^ g^–1^. Such a favorable textural structure is desirable for catalytic applications.^23^,^25^,^30^

### Characterization of Co dispersion

**Fig. 1:**
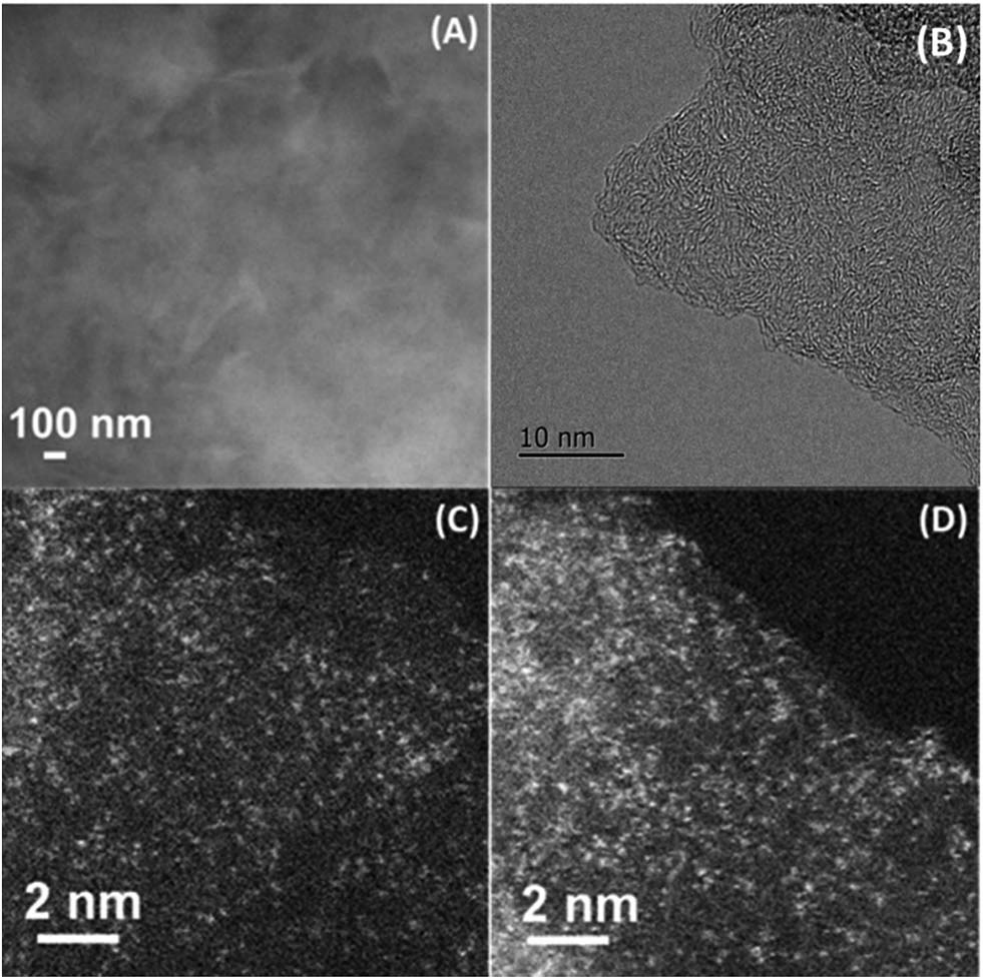
SEM (A), HRTEM (B), and HAADF-STEM (C, D) images of Co–N–C catalyst. The white dots in (C, D) are Co single atoms.

The dispersion of the Co–N–C catalyst was determined with both XRD and electron microscopy techniques. First, XRD patterns (Fig. S3†) did not show any peaks assignable to cobalt metal or its compounds, suggesting that the Co species in the sample are either highly dispersed or amorphous. Then, normal electron microscopy techniques including SEM, STEM and HRTEM were employed to examine the dispersion of Co species. In good agreement with the XRD results no cobalt-containing nanoparticles were observed, but graphitic layers were revealed by our efforts to examine many different areas of the sample (Fig. 1A and B, and more images see Fig. S4 and S5†), implying that the cobalt species must be highly dispersed as tiny clusters or single atoms that are undetectable or invisible by XRD, SEM and HRTEM techniques. Meanwhile, the elemental mapping result (Fig. S6†) indicates that the signals of Co, N and C are completely superimposed on each other, at least on the nanoscale, suggesting that Co might be bonded with N or C. In order to provide information at an atomic scale, we then used the sub-Ångström-resolution HAADF-STEM technique to inspect the Co–N–C catalyst. To our surprise, a large number of uniformly dispersed Co single atoms were clearly observed (Fig. 1C and D); examination of different regions revealed that no clusters or small particles were present in the vicinity of the single atoms (more images see Fig. S7†). Assuming that all the Co components in the sample are dispersed as single atoms, we calculated the density of Co atoms to be approximately 0.55 Co atoms per nm^2^ based on the BET surface area and the loading of cobalt. Such a high density was rarely obtained in supported precious metal SACs.^17^,^19^,^24^

### Structure identification of Co–N–C

**Fig. 2:**
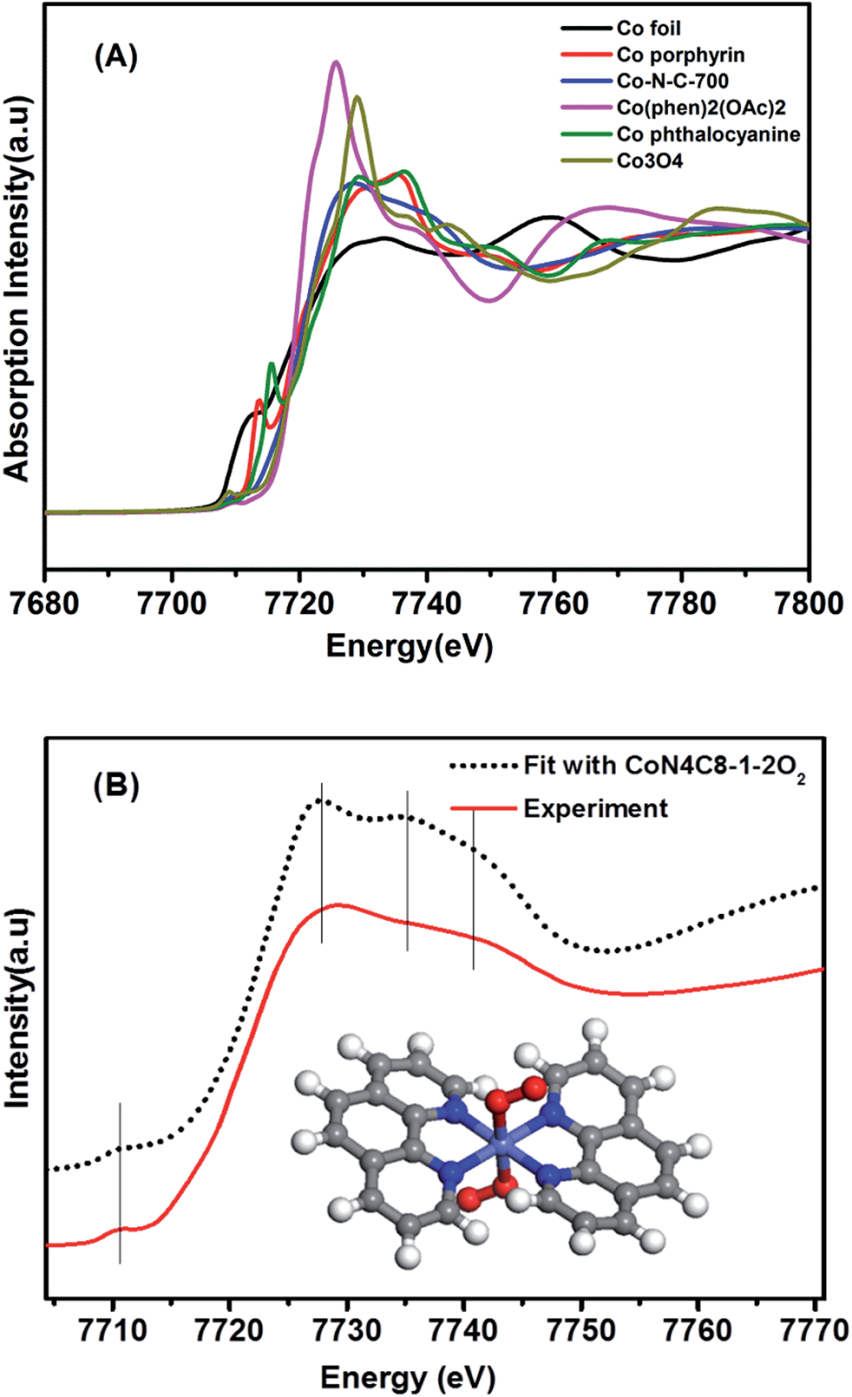
(A) The normalized XANES spectra at the Co K-edge of different samples. (B) Comparison between the K-edge XANES experimental spectrum of Co–N–C (solid red line) and the theoretical spectrum (black dotted line) calculated with the inset structure.

Having this single-atom dispersed Co–N–C catalyst in hand, we are able to identify the structure associated with Co atoms. It has been well established that XAS is a powerful technique to determine the chemical state and coordination environment of the centre atoms in the sample.^31^,^32^ Fig. 2A shows the XANES spectra at the Co K edge of the Co–N–C sample as well as of several reference samples. In comparison with both Co(ii) phthalocyanine (green line) and Co(ii) porphyrin (red line) complexes featuring a pre-edge peak at 7714–7716 eV (which was regarded as a fingerprint of Co–N_4_ square-planar structures), the absence of a pre-edge peak in the Co–N–C catalyst (blue line) suggested that it was not a planar structure.^33^,^34^ Likewise, the catalyst precursor Co(phen)_2_(OAc)_2_ also lacked a pre-edge peak due to the tetragonal-like structure of Co(ii) phenanthroline. A comparison of the *E*_0_ value (the first inflection point) showed that the Co–N–C sample had almost the same *E*_0_ value (7720 eV) as its precursor (Table S1†), which was assigned to Co^2+^, whereas the reference Co foil and Co_3_O_4_ had *E*_0_ values of 7709 eV and 7727 eV, respectively. Further examination of Co–N–C samples which were obtained at different annealing temperatures showed that the pyrolysis at 900 °C resulted in the formation of metallic Co while that at 500–700 °C always yielded Co^2+^ (Table S1†).

To determine the structure of the Co–N–C sample, we then used DFT to establish various architectural models based on Co(ii) porphyrin with or without considering coordination with oxygen atoms in the axial direction (Table S2†). Firstly, we calculated the XANES spectra of the reference sample, Co porphyrin, to verify the validity of our calculation method. As shown in Fig. S8,† the calculated result was in good agreement with the experimental spectrum of the Co porphyrin, validating our calculation method. Then, with those Co–N–C models, we calculated XANES (dotted lines in Fig. S9†) and found that the calculation results for CoN_4_C_8_-1-2O_2_ could best reproduce the main features of the experimental spectrum, as shown in Fig. 2B. In this structure, the Co center atom is coordinated with four pyridinic N atoms, while the whole structure is deformed from a plane. Perpendicular to the Co–N_4_ plane, two oxygen atoms are weakly adsorbed on the Co center. In comparison with the Fe–N–C structure (FeN_4_C_12_O_2_) reported by Zitolo *et al.*,^34^ the present Co–N–C catalyst adopted a quite different structure, which may result from an intrinsic difference in the electronic properties between Fe and Co. Such a different structure may also account for their different reactivities in both electrocatalysis and chemoselective transformation of organic molecules.^2^,^4^,^5^,^35^

**Fig. 3:**
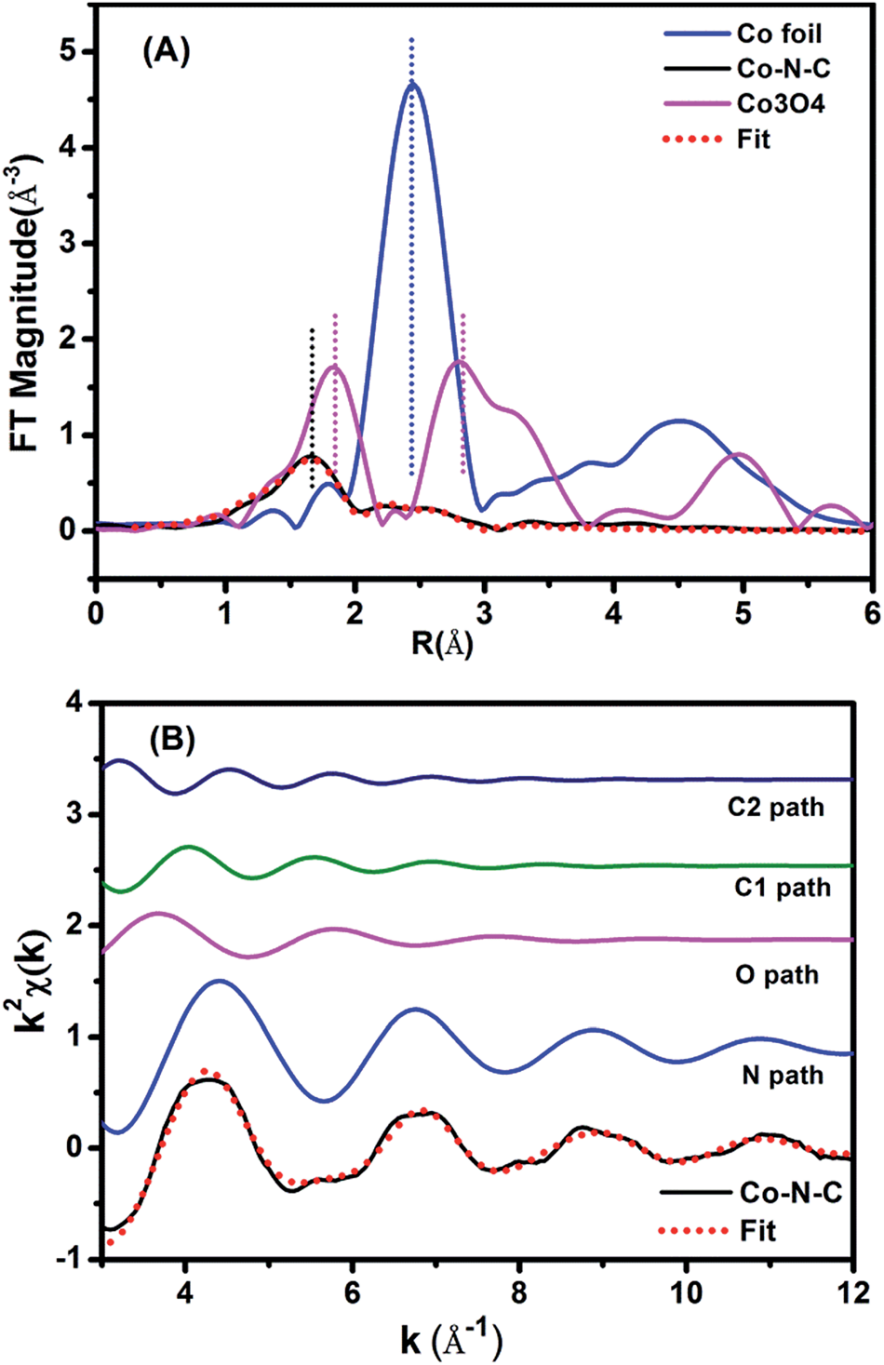
(A) The *k*^2^-weighted Fourier transform spectra of the experimental and fitted Co–N–C catalyst as well as the Co foil and Co_3_O_4_ reference samples. (B) The contributions of different paths including Co–N (blue line), Co–O (pink line) and Co–C (green and navy blue lines) in *k*-space for the Co–N–C sample.

The above CoN_4_C_8_-1-2O_2_ structure was further corroborated with EXAFS analysis. Fig. 3A shows the Fourier-transformed *k*^2^-weighted EXAFS spectra at the Co K edge. In contrast to the reference samples, Co foil and Co_3_O_4_, our Co–N–C catalyst did not present a prominent peak at the positions of either Co–Co or Co–O coordination, supporting the conclusion that it does not contain either metallic Co or CoO_*x*_ species. The EXAFS data were further fitted with the model in Fig. 2B in *r*-space (Fig. 3A), *k*-space (Fig. 3B), and *q*-space (Fig. S10†) in the ranges of Δ*r* (1.1–3.5 Å) and Δ*k* (3.0–12.0 Å^–1^), respectively, and the fitted results are in good agreement with the experimental ones, corroborating the structure of CoN_4_C_8_-1-2O_2_. Moreover, the oscillation of Co–N is the most intense, indicating that Co–N is the nearest shell close to the center and contributes the most to the total oscillations. The best-fit result of the EXAFS data is summarized in Table 1, showing the Co–N shell with a coordination number (CN) of 3.7 at a distance of 1.88 Å, the Co–O shell with a CN of 1.7 at a distance of 2.08 Å, and the Co–C shell with a CN of 4.0 and 4.2 at a distance of 2.76 Å and 3.21 Å, respectively. This result is found to be consistent, within an acceptable error, with the structural parameters of CoN_4_C_8_-1-2O_2_ determined by DFT and XANES calculations (Table S2†).

**Table 1:** EXAFS data fitting results of Co–N–C catalyst^*a*^

Sample	Shell	*N*	*R*/Å	*σ* ^2^ × 10^2^/Å^2^	Δ*E*_0_/eV	*r*-Factor (%)
Co–N–C	Co–N	3.7	1.88	0.7	–7.9	0.29
	Co–O	1.7	2.08	1.6	–7.9	
	Co–C_1_	4.0	2.76	1.8	–7.9	
	Co–C_2_	4.2	3.21	1.8	–7.9	

^*a*^ *N*, the coordination number for the absorber–backscatterer pair. *R*, the average absorber–backscatterer distance. *σ*^2^, the Debye–Waller factor. Δ*E*_0_, the inner potential correction. The accuracies of the above parameters were estimated as *N*, ±20%; *R*, ±1%; *σ*^2^, ±20%; Δ*E*_0_, ±20%. The data range used for data fitting in *k*-space (Δ*k*) and *r*-space (Δ*r*) are 3.0–12.0 Å^–1^ and 1.1–3.5 Å, respectively.

### XPS characterization

**Fig. 4:**
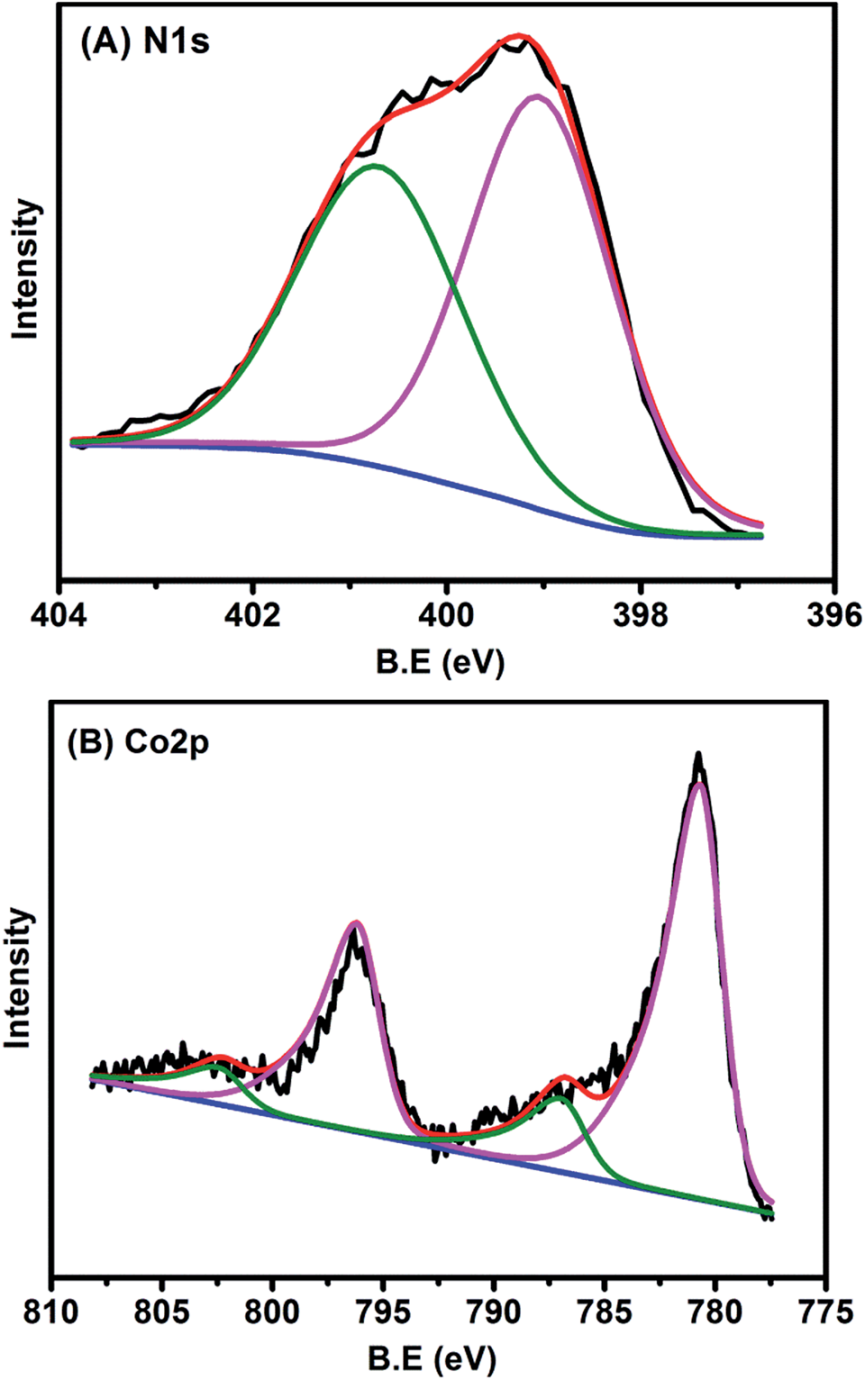
(A) N 1s and (B) Co 2p XPS spectra of Co–N–C catalyst.

The chemical states of Co and N in the sample were also examined with XPS. The N 1s spectrum (Fig. 4A) could be deconvoluted into two peaks centered at the binding energy of 400.8 and 399.1 eV, which were assigned to graphitic and pyridinic N, respectively.^2^,^36^ The formation of graphitic N was due to the graphitization of the precursor 1,10-phenanthroline that was not coordinated with Co cations, while the predominance of pyridinic N (accounting for 57.5% in total N species) resulted from coordination with cobalt, according to the structure in Fig. 2B. In agreement with the implications of XPS of N 1s, the XPS spectrum of Co 2p_3/2_ is characteristic of Co^2+^ species (Fig. 4B), as indicated by the binding energy of 780.7 eV and the presence of a satellite peak (green line).^37^ The predominance of Co^2+^ was also supported by electron paramagnetic resonance (EPR) experiments (Fig. S11†).^38^ This Co^2+^ species, strongly coordinated with four N atoms in the graphitic layer, is robust enough to resist against either aggregation at high temperatures or leaching by acid treatment. In addition to N and Co, there were also C (79.26 at%), O (11.36 at%), and the residue Mg (0.06 at%) detected by XPS on the catalyst surface (Table S3 and Fig. S12†). It should be highlighted that the atomic concentration of Co determined by XPS was 0.7 at%, corresponding to a weight percentage of 3.5 wt%, which was very close to the ICP-AES analysis result (3.6 wt%), demonstrating the homogeneous dispersion of Co throughout the whole material. The O 1s spectrum was deconvoluted into two peaks at 531.5 and 533.1 eV, which were ascribed to ketonic C=O groups and C–O groups, respectively (Fig. S12†). The absence of a Co–O peak in the O 1s was probably due to the removal of weakly adsorbed oxygen molecules when the sample was brought into high vacuum to record the XPS spectra.

### Catalytic performance

**Fig. 5:**
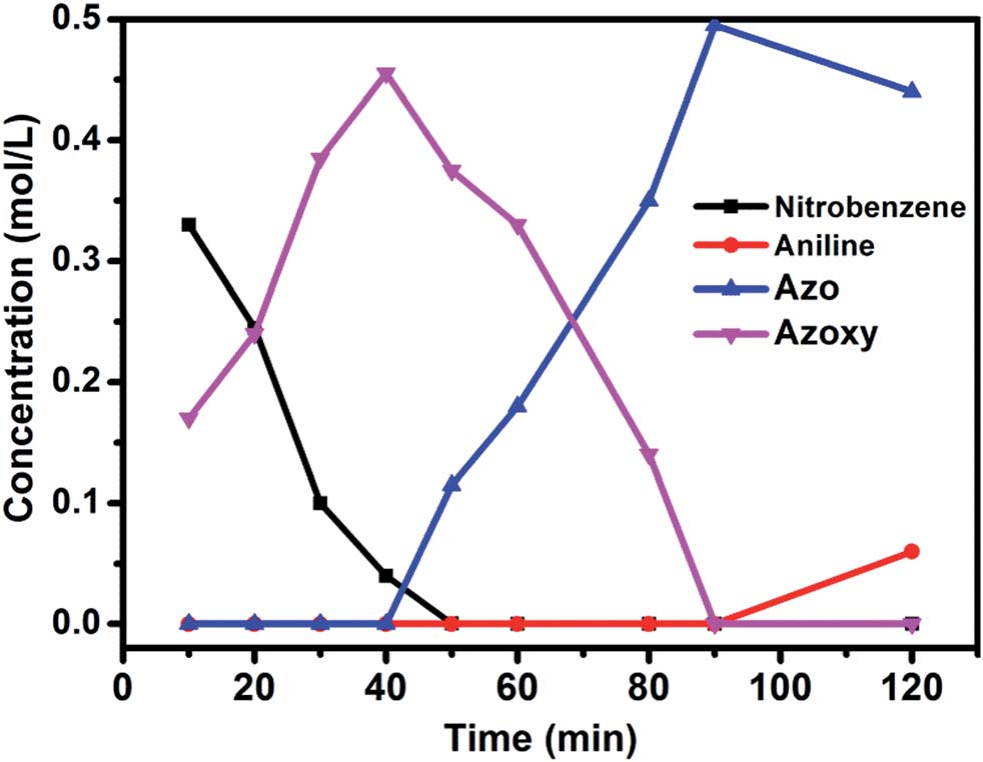
Substrate and product concentration profiles as a function of the reaction time. Reaction conditions: Co–N–C catalyst: 0.7 mol%; NaOH (0.2 equiv.); solvent: *t*-BuOH (2 mL); 80 °C; 3 MPa H_2_.

The well-defined and atomically dispersed Co–N–C catalyst was evaluated in the hydrogenation of nitroarenes to azo compounds, an important reaction in the fields of dyes and pharmaceuticals.^39^–^46^ Investigation of the reaction conditions (Table S4†) showed that both the activity and selectivity were significantly affected by the reaction temperature and time, H_2_ pressure, solvent, and the addition of base. Under the optimized reaction conditions (80 °C, 3 MPa H_2_, 1.5 h, *tert*-butyl alcohol as the solvent, catalyst loading of 0.7 mol% Co, 0.2 equivalent of NaOH), the isolated yield of azo reached 97%. The kinetic results (Fig. 5) showed that in the beginning 40 min nitrobenzene was reduced to azoxybenzene which was then further reduced to azobenzene in the following 50 min and aniline with extended duration. Clearly, azoxy was an intermediate for the azo product, consistent with the earlier proposed nitro–nitroso–hydroxylamine condensation pathway (Fig. S13†).^40^,^47^ In this tandem reaction the Co–N–C catalyst was believed to catalyze the hydrogenation steps whereas a small amount of alkali (NaOH) was required to catalyze the condensation step between nitrosobenzene and hydroxylamine. As the condensation step was too fast to be observed, the hydrogenation step appeared as a rate-determining step. It was also found that the reaction rate was first-order dependence on H_2_ pressure (Fig. S14†), suggesting that hydrogen dissociation on the Co–N–C active sites proceed smoothly.^48^ The calculation of the turnover frequency (TOF) based on the initial conversion of nitrobenzene per Co atom showed that the Co–N–C catalyst afforded a TOF of 35.9 h^–1^, which was only slightly lower than that over precious metals (*e.g.*, TOF over Au/Mg_4_Al was 41.8 h^–1^),^44^ demonstrating the potential of the Co–N–C material to replace expensive noble metal catalysts in chemical transformations.

What is of more interest is the unprecedented selectivity of the Co–N–C catalyst for diverse nitroarene substrates, particularly those bearing reducible groups. As shown in Table 2, both the electron-rich (Table 2, entries 2–6) and electron-deficient (Table 2, entries 7–12) nitroarenes were converted quickly and selectively into the corresponding azo products. Moreover, for the more challenging substrate 3-nitrostyrene (Table 2, entry 6), the catalyst could reduce the nitro groups without any detectable concurrent reduction of the unsaturated alkenes. Both the control reaction and the ATR-IR (Attenuated Total Reflection-Infrared) spectroscopy results (Table S5 and Fig. S15†) revealed that the alkene group could not be adsorbed on the Co–N–C moieties at all, which should account for the extraordinarily high selectivity. Interestingly, halogenated nitroarenes (Table 2, entries 8–12) can also be excellent and useful substrates for selective hydrogenative coupling reactions. Both mono and dihalogenated nitroarenes were all quickly converted into the corresponding chloro, bromo, or dihalogenated azo compounds without other by-products. Notably, for aryl iodides (Table 2, entry 12) that are prone to undergo dehalogenation on many precious catalysts, the yield of the iodo azo compound on Co–N–C catalysts reached 94% without dehalogenation. Furthermore, the catalyst could be easily recovered from the reaction system by filtration and could be reused for at least 5 times without change in selectivity or yield, provided that the reaction time was suitably extended (Table S6†). HAADF-STEM images showed that the Co single atoms were still uniformly dispersed throughout the sample after reuse and no aggregation was observed (Fig. S16†), demonstrating the high stability of the Co–N–C catalyst.

**Table 2:** Substrate scope of the hydrogenation reaction^*a*^

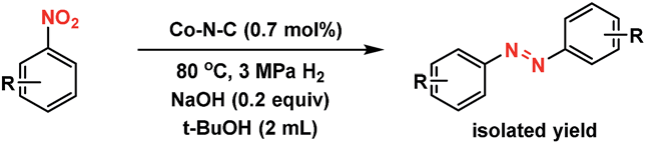
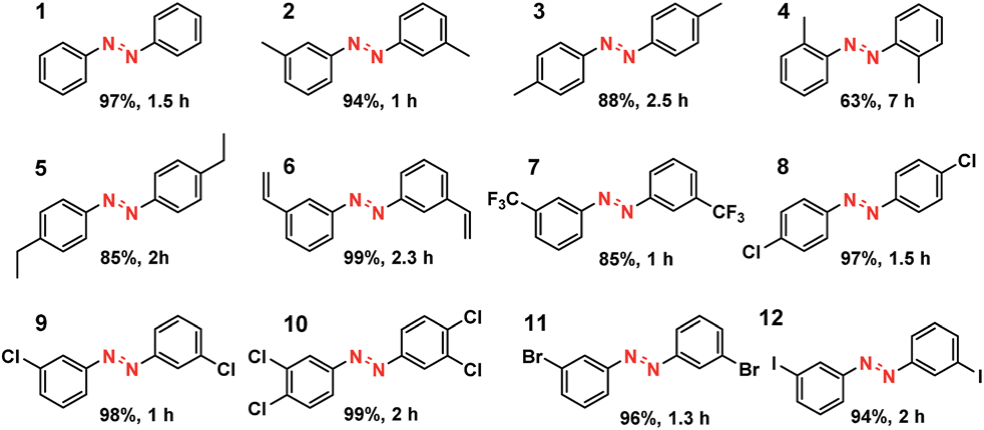

^*a*^For detailed reaction conditions, refer to the ESI.† Yields refer to isolated products.

## Conclusions

In summary, we have successfully synthesized self-supporting atomically dispersed Co–N–C catalysts. The exact structure of the catalyst was identified to be CoN_4_C_8_-1-2O_2_ where Co a single atom was strongly coordinated with four pyridinic nitrogen atoms within graphitic layers, and oxygen atoms were weakly adsorbed on the Co atoms perpendicular to the Co–N_4_ plane. Such a unique structure showed excellent activity, chemoselectivity and stability for the synthesis of aromatic azo compounds through hydrogenative coupling of nitroarenes, thus providing new opportunities of using earth-abundant elements to replace scarce and high-cost noble metal catalysts in a variety of chemical transformations. More extensively, with the identification of the catalytically active structure, design and synthesis of the better performance M–N–C catalysts will be expected in future, facilitating the real replacement of platinum in both thermo- and electro-catalysis in practice.

## Supplementary Material

Supplementary information

## References

[cit1] Masa J., Xia W., Muhler M., Schuhmann W. (2015). Angew. Chem., Int. Ed..

[cit2] Liang H. W., Wei W., Wu Z. S., Feng X., Mullen K. (2013). J. Am. Chem. Soc..

[cit3] Liang Y., Li Y., Wang H., Zhou J., Wang J., Regier T., Dai H. (2011). Nat. Mater..

[cit4] Lefevre M., Proietti E., Jaouen F., Dodelet J. P. (2009). Science.

[cit5] Wu G., More K. L., Johnston C. M., Zelenay P. (2011). Science.

[cit6] Tran P. D., Morozan A., Archambault S., Heidkamp J., Chenevier P., Dau H., Fontecave M., Martinent A., Jousselme B., Artero V. (2015). Chem. Sci..

[cit7] McGuire Jr R., Dogutan D. K., Teets T. S., Suntivich J., Shao-Horn Y., Nocera D. G. (2010). Chem. Sci..

[cit8] Zhang L., Wang A., Wang W., Huang Y., Liu X., Miao S., Liu J., Zhang T. (2015). ACS Catal..

[cit9] Westerhaus F. A., Jagadeesh R. V., Wienhofer G., Pohl M. M., Radnik J., Surkus A. E., Rabeah J., Junge K., Junge H., Nielsen M., Bruckner A., Beller M. (2013). Nat. Chem..

[cit10] Wei Z., Wang J., Mao S., Su D., Jin H., Wang Y., Xu F., Li H., Wang Y. (2015). ACS Catal..

[cit11] Zhong W., Liu H., Bai C., Liao S., Li Y. (2015). ACS Catal..

[cit12] Cheng T., Yu H., Peng F., Wang H., Zhang B., Su D. (2016). Catal. Sci. Technol..

[cit13] Yang C., Fu L., Zhu R., Liu Z. (2016). Phys. Chem. Chem. Phys..

[cit14] Li Y., Zhou W., Wang H., Xie L., Liang Y., Wei F., Idrobo J. C., Pennycook S. J., Dai H. (2012). Nat. Nanotechnol..

[cit15] Deng D., Yu L., Chen X., Wang G., Jin L., Pan X., Deng J., Sun G., Bao X. (2013). Angew. Chem., Int. Ed..

[cit16] Zhu Y., Zhang B., Liu X., Wang D. W., Su D. S. (2014). Angew. Chem., Int. Ed..

[cit17] Jin H., Wang J., Su D., Wei Z., Pang Z., Wang Y. (2015). J. Am. Chem. Soc..

[cit18] Liang H. W., Bruller S., Dong R., Zhang J., Feng X., Mullen K. (2015). Nat. Commun..

[cit19] Wu Z. Y., Xu X. X., Hu B. C., Liang H. W., Lin Y., Chen L. F., Yu S. H. (2015). Angew. Chem., Int. Ed..

[cit20] Jagadeesh R. V., Surkus A. E., Junge H., Pohl M. M., Radnik J., Rabeah J., Huan H., Schunemann V., Bruckner A., Beller M. (2013). Science.

[cit21] Yang X., Wang A., Qiao B., Li J., Liu J., Zhang T. (2013). Acc. Chem. Res..

[cit22] Lin J., Wang A., Qiao B., Liu X., Yang X., Wang X., Liang J., Li J., Liu J., Zhang T. (2013). J. Am. Chem. Soc..

[cit23] Qiao B., Wang A., Yang X., Allard L. F., Jiang Z., Cui Y., Liu J., Li J., Zhang T. (2011). Nat. Chem..

[cit24] Wei H., Liu X., Wang A., Zhang L., Qiao B., Yang X., Huang Y., Miao S., Liu J., Zhang T. (2014). Nat. Commun..

[cit25] Kyriakou G., Boucher M. B., Jewell A. D., Lewis E. A., Lawton T. J., Baber A. E., Tierney H. L., Flytzani-Stephanopoulos M., Sykes E. C. H. (2012). Science.

[cit26] Pei G. X., Liu X. Y., Wang A., Lee A. F., Isaacs M. A., Li L., Pan X., Yang X., Wang X., Tai Z., Wilson K., Zhang T. (2015). ACS Catal..

[cit27] Yan H., Cheng H., Yi H., Lin Y., Yao T., Wang C., Li J., Wei S., Lu J. (2015). J. Am. Chem. Soc..

[cit28] Lucci F. R., Liu J., Marcinkowski M. D., Yang M., Allard L. F., Flytzani-Stephanopoulos M., Sykes E. C. (2015). Nat. Commun..

[cit29] Fei H., Dong J., Arellano-Jimenez M. J., Ye G., Dong Kim N., Samuel E. L., Peng Z., Zhu Z., Qin F., Bao J., Yacaman M. J., Ajayan P. M., Chen D., Tour J. M. (2015). Nat. Commun..

[cit30] Vilé G., Albani D., Nachtegaal M., Chen Z., Dontsova D., Antonietti M., López N., Pérez-Ramírez J. (2015). Angew. Chem., Int. Ed..

[cit31] Mutz B., Carvalho H. W. P., Mangold S., Kleist W., Grunwaldt J.-D. (2015). J. Catal..

[cit32] Gänzler A. M., Casapu M., Boubnov A., Müller O., Conrad S., Lichtenberg H., Frahm R., Grunwaldt J.-D. (2015). J. Catal..

[cit33] Ramaswamy N., Tylus U., Jia Q., Mukerjee S. (2013). J. Am. Chem. Soc..

[cit34] Zitolo A., Goellner V., Armel V., Sougrati M. T., Mineva T., Stievano L., Fonda E., Jaouen F. (2015). Nat. Mater..

[cit35] Zhao Y., Watanabe K., Hashimoto K. (2012). J. Am. Chem. Soc..

[cit36] Jaouen F., Herranz J., Lefevre M., Dodelet J. P., Kramm U. I., Herrmann I., Bogdanoff P., Maruyama J., Nagaoka T., Garsuch A., Dahn J. R., Olson T., Pylypenko S., Atanassov P., Ustinov E. A. (2009). ACS Appl. Mater. Interfaces.

[cit37] Singh D., Soykal I. I., Tian J., von Deak D., King J., Miller J. T., Ozkan U. S. (2013). J. Catal..

[cit38] Antolini E., Zhecheva E. (1998). Mater. Lett..

[cit39] Haber F. (1898). Z. Elektrochem..

[cit40] Blaser H. U. (2006). Science.

[cit41] Corma A., Concepcion P., Serna P. (2007). Angew. Chem., Int. Ed..

[cit42] Grirrane A., Corma A., García H. (2008). Science.

[cit43] Merino E. (2011). Chem. Soc. Rev..

[cit44] Liu X., Li H. Q., Ye S., Liu Y. M., He H. Y., Cao Y. (2014). Angew. Chem., Int. Ed..

[cit45] Cai S., Duan H., Rong H., Wang D., Li L., He W., Li Y. (2013). ACS Catal..

[cit46] Yang H., Cui X., Deng Y., Shi F. (2013). ChemCatChem.

[cit47] Zhou B., Song J., Zhou H., Wu T., Han B. (2016). Chem. Sci..

[cit48] Ge H., Zhang B., Gu X., Liang H., Yang H., Gao Z., Wang J., Qin Y. (2016). Angew. Chem., Int. Ed..

