# Supplementary material for: Single-atom dispersed Co–N–C catalyst: structure identification and performance for hydrogenative coupling of nitroarenes
Source: Chem Sci. 2016 Jun 13;7(9):5758–64. doi: 10.1039/c6sc02105k (PMC6021980; doi:10.1039/c6sc02105k)
Supplement: Supplementary file 1 [file SC-007-C6SC02105K-s001.pdf]

Electronic Supplementary Material (ESI) for Chemical Science.
This journal is © The Royal Society of Chemistry 2016

Supporting Information

Single-atom dispersed Co-N-C catalyst: structure identification and performance for hydrogenative coupling of nitroarenes

Wengang Liu[a,b,‡], Leilei Zhang[a,‡], Wensheng Yan[c], Xiaoyan Liu[a], Xiaofeng Yang[a], Shu Miao[a], Wentao Wang[a], Aiqin Wang[a,*], Tao Zhang[a,*]

[a] State Key Laboratory of Catalysis, *i*ChEM (Collaborative Innovation Center of Chemistry for Energy Materials), Dalian Institute of Chemical Physics, Chinese Academy of Sciences, Dalian, 116023, China.

[b] University of Chinese Academy of Sciences, Beijing 100049, China.

[c] National Synchrotron Radiation Laboratory, University of Science and Technology of China, Hefei, 230029, China

‡These authors contributed equally to this work.

* Correspondence to: aqwang@dicp.ac.cn; taozhang@dicp.ac.cn

1. **Characterization of the Co-N-C catalyst.**

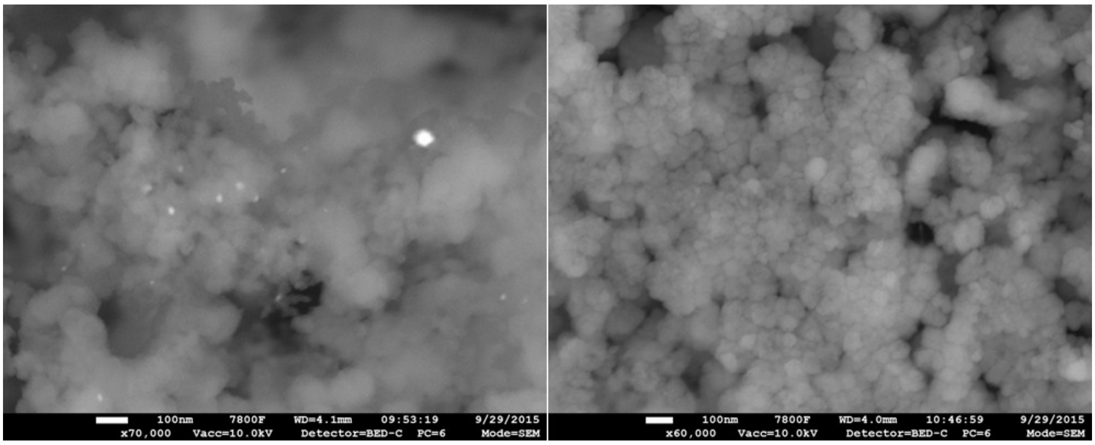

Figure S1. Representative SEM images of Co-N-C/Carbon (left) and Co-N-C/MgO (right) catalysts under back-scattering electron detector mode. The bright spots in the left image are big Co particles due to aggregation; in contrast, they are absent in the right image, indicating MgO support effectively limited the aggregation of cobalt particles.

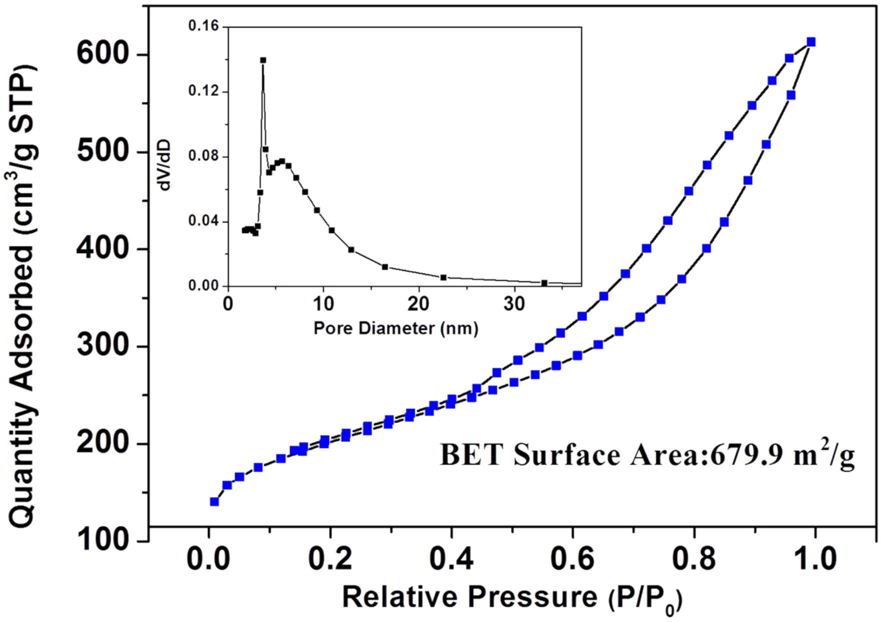

Figure S2. N_2 adsorption-desorption isotherms of Co-N-C catalyst. Inset is the mesopore size distribution.

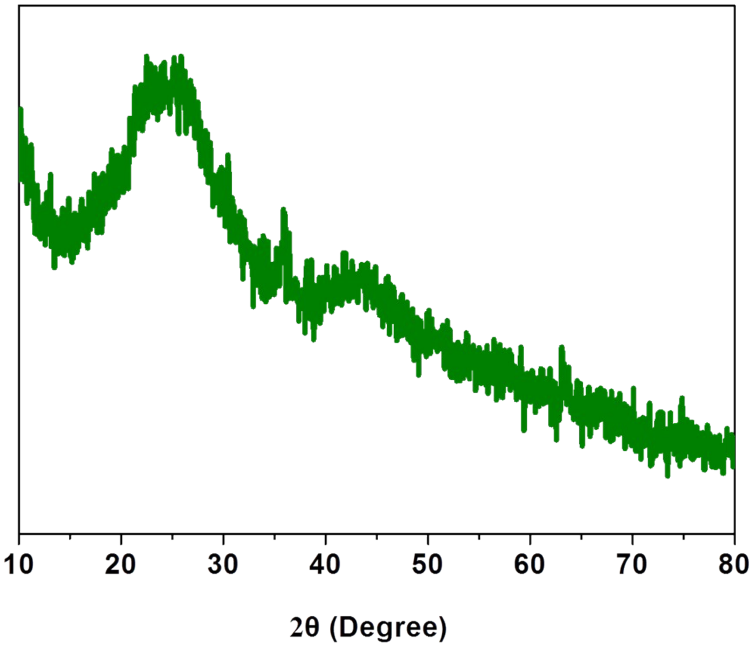

Figure S3. XRD pattern of the Co-N-C catalyst. The broad peaks located at 23° and 43° could be ascribed to the (002) and (004) planes of carbon matrix. No diffraction patterns of Co/CoO_x can be observed.

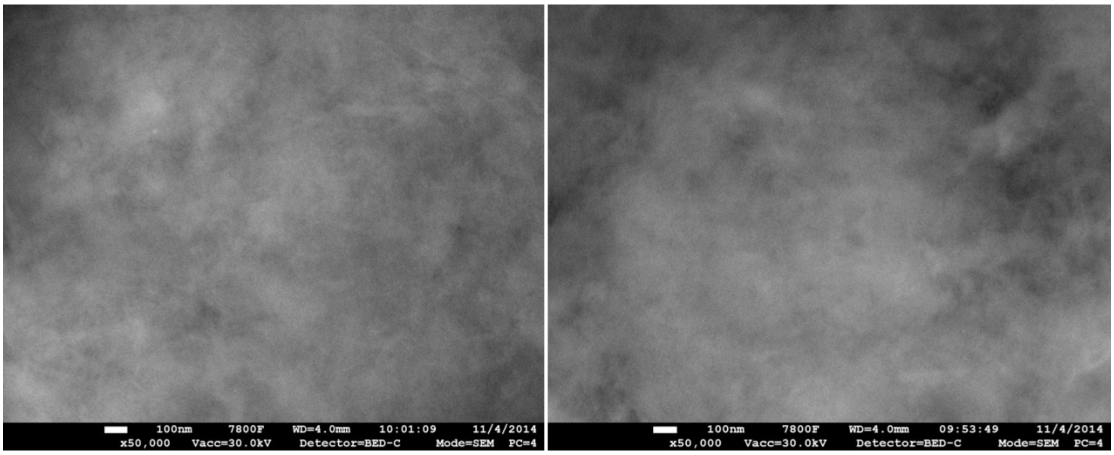

Figure S4. SEM images of Co-N-C catalyst under back-scattering electron detector mode.

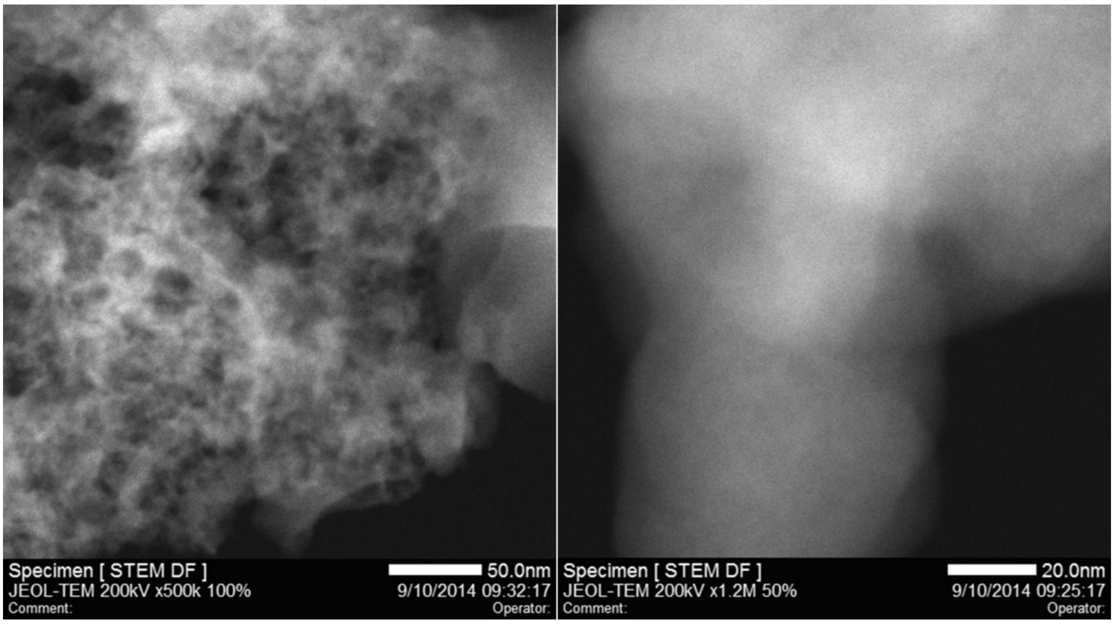

Figure S5. STEM images of Co-N-C catalyst

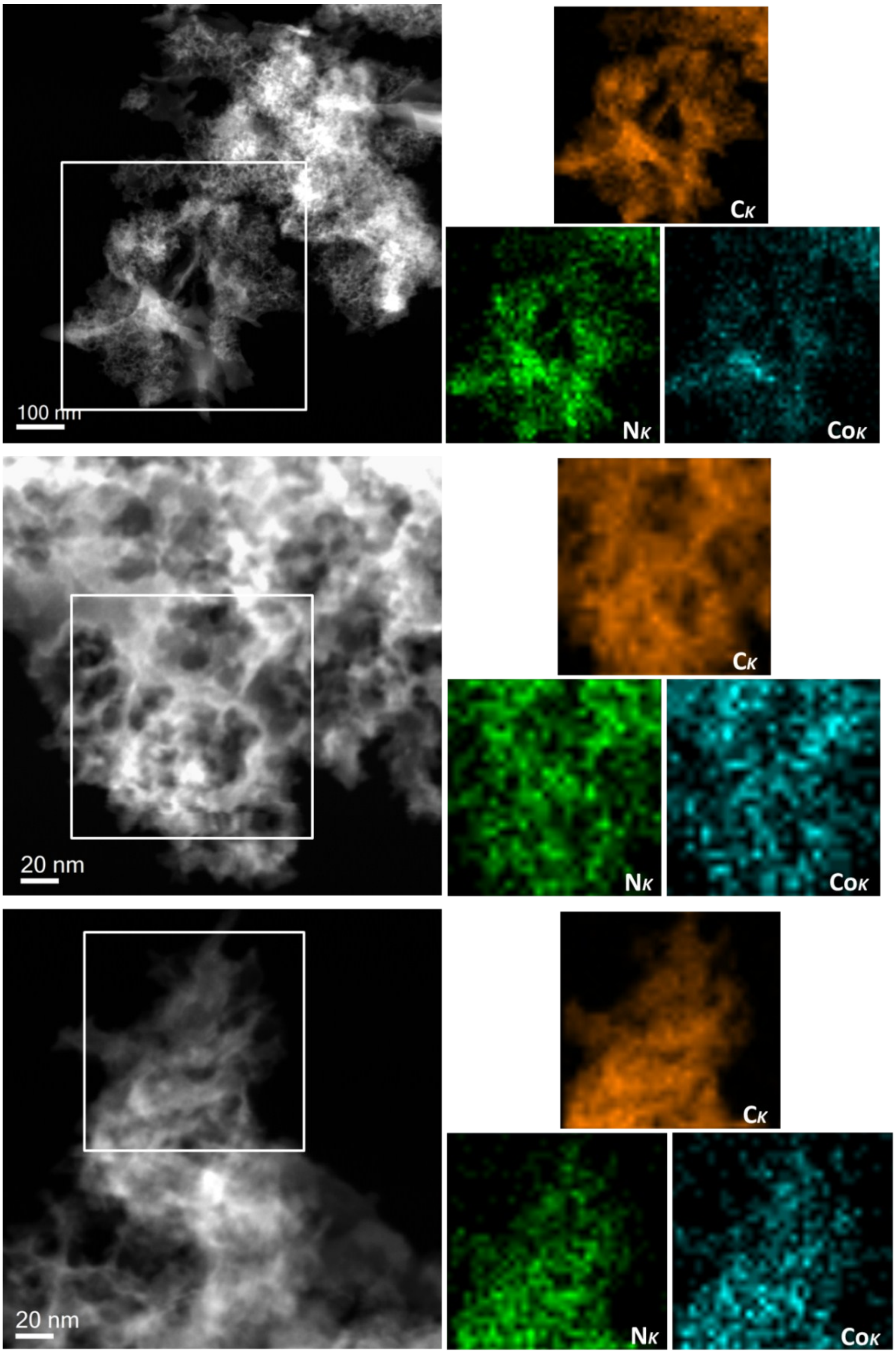

Figure S6. Elemental mapping of Co-N-C catalyst in different regions.

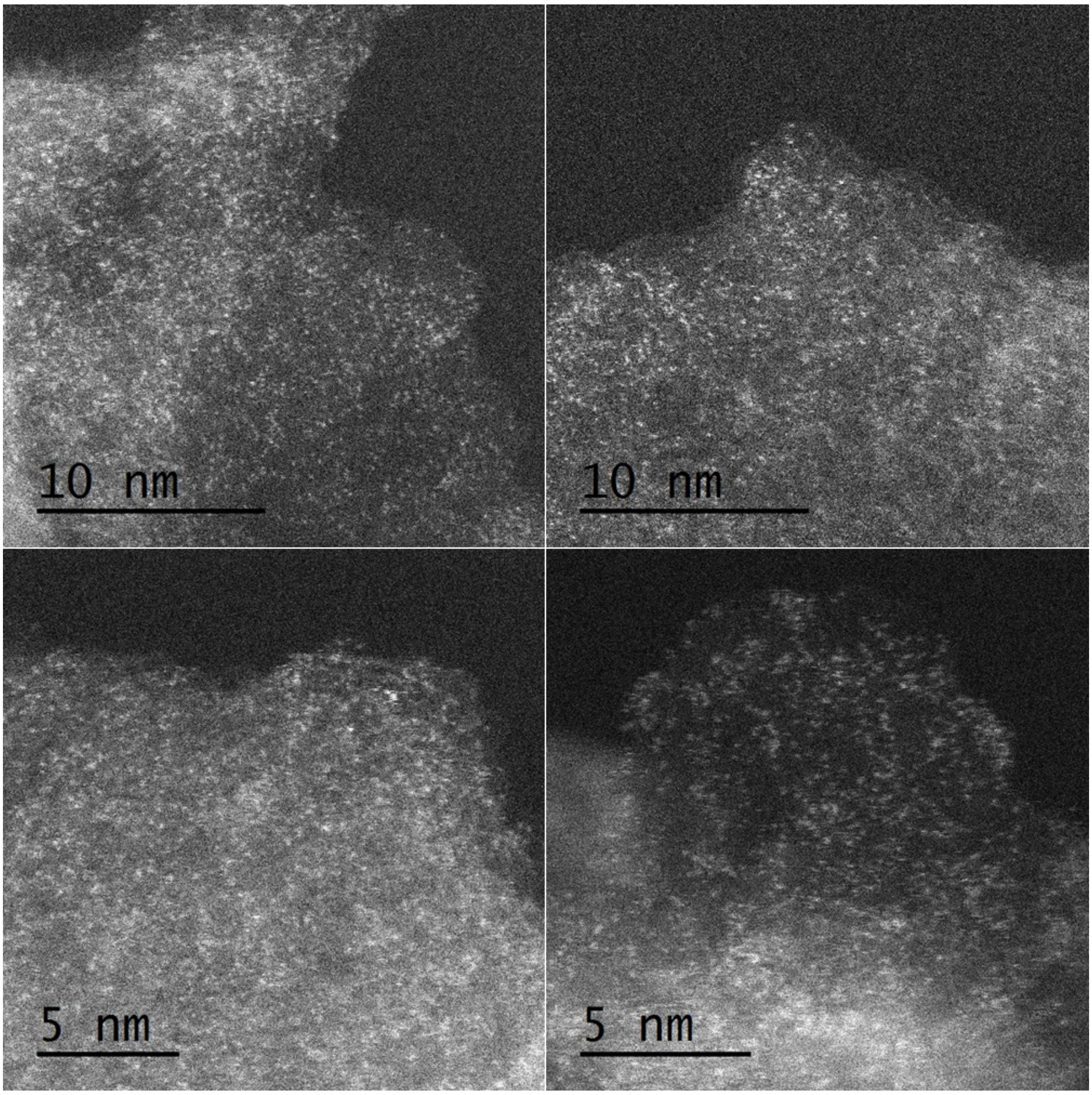

Figure S7. Subångström-resolution HAADF-STEM images of different regions of Co-N-C catalyst. The white dots in images are Co single atoms.

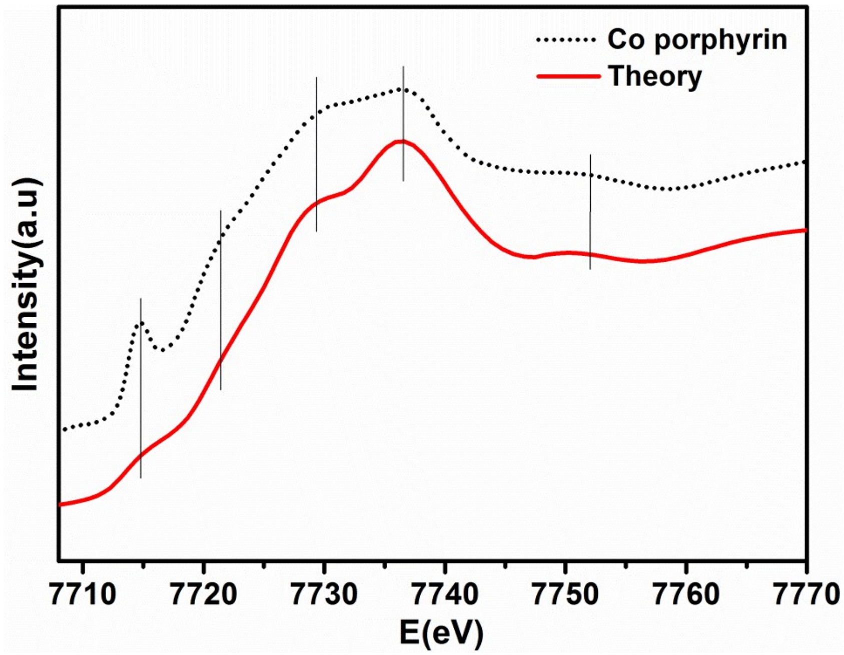

Figure S8. Comparison between the XANES experimental spectrum of Co porphyrin at Co K edge (solid red lines) and the theoretical spectra calculated with the depicted structures (black dashed lines).

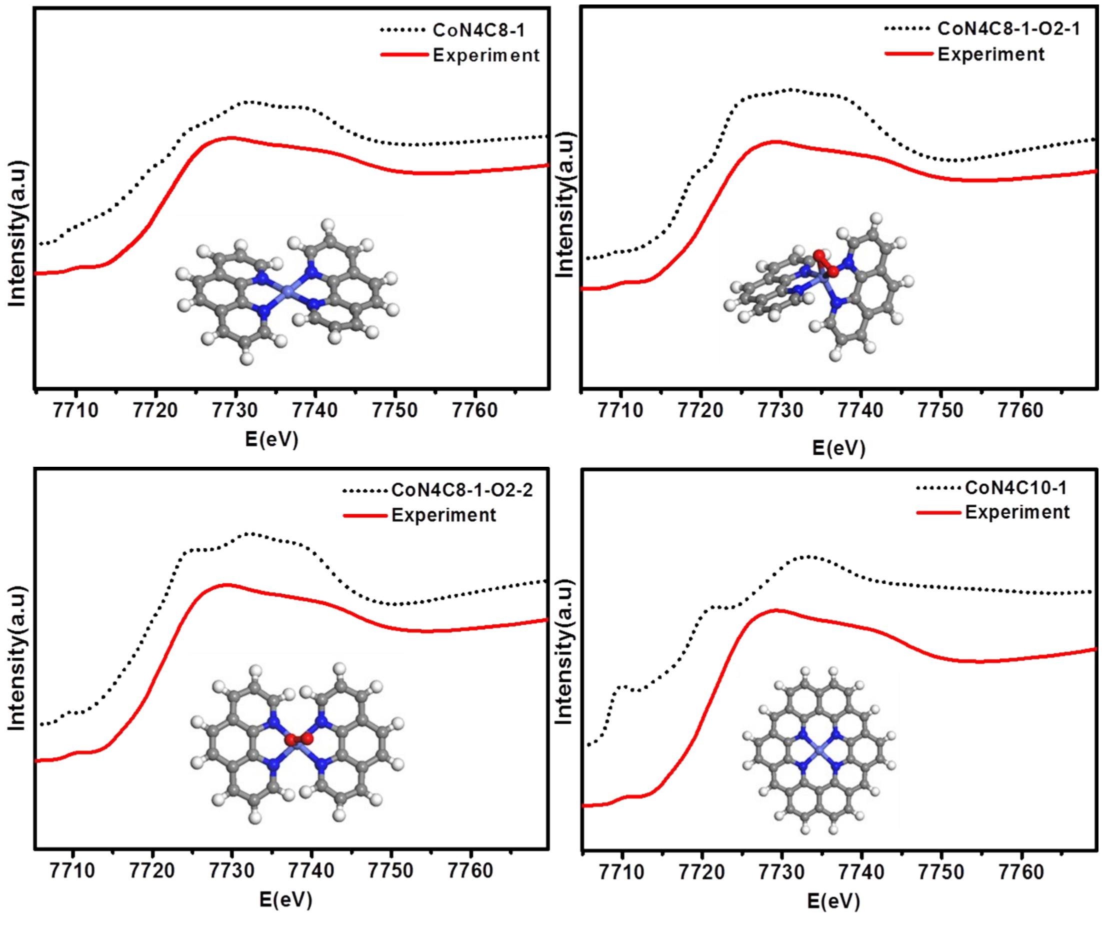

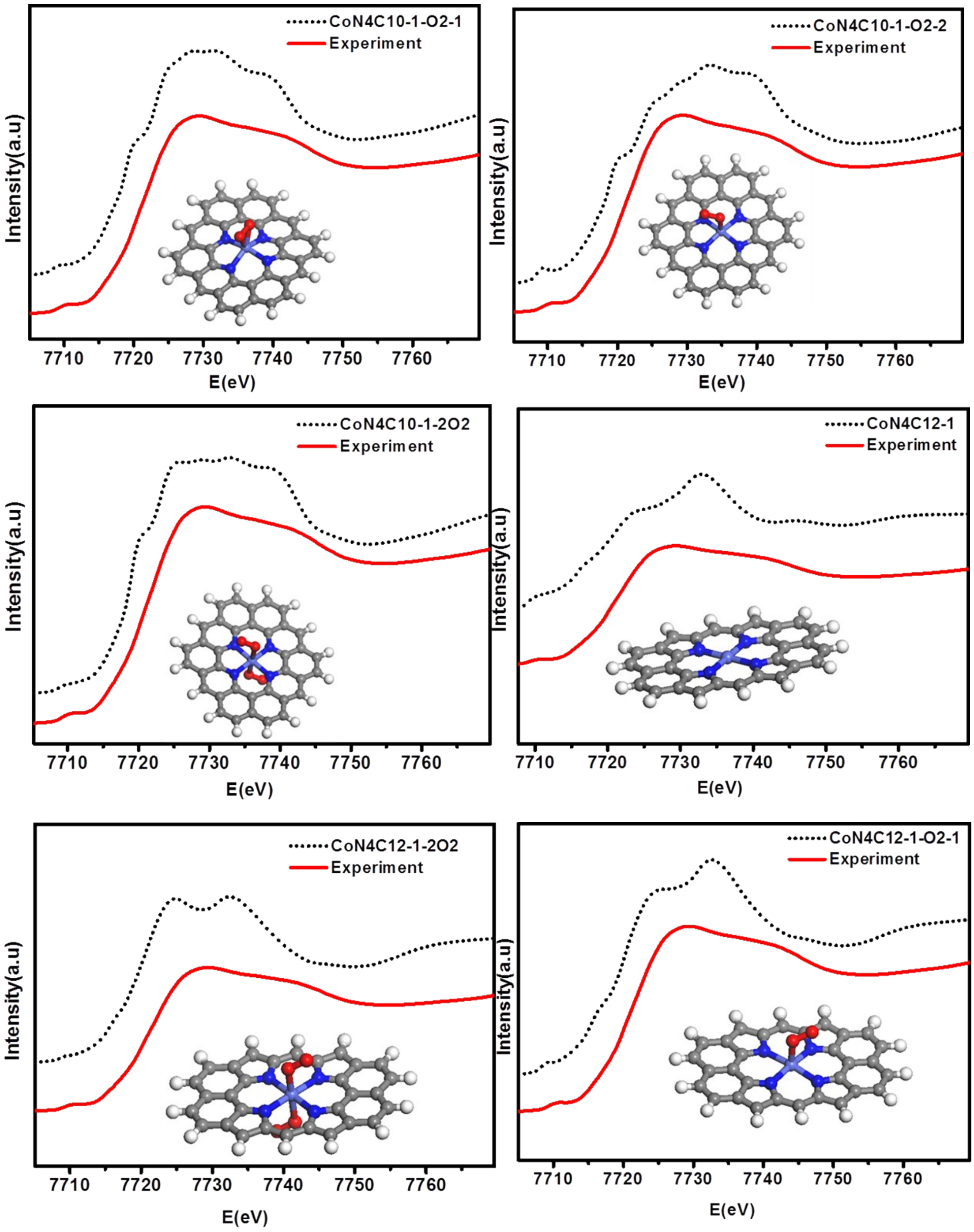
CoN4C10-1-O2-1
Experiment
Intensity(a.u)
7710
7720
7730
7740
7750
7760
E(eV)
CoN4C10-1-O2-2
Experiment
Intensity(a.u)
E(eV)
CoN4C10-1-2O2
Experiment
Intensity(a.u)
E(eV)
CoN4C12-1
Experiment
Intensity(a.u)
E(eV)
CoN4C12-1-2O2
Experiment
Intensity(a.u)
E(eV)
CoN4C12-1-O2-1
Experiment
Intensity(a.u)
E(eV)

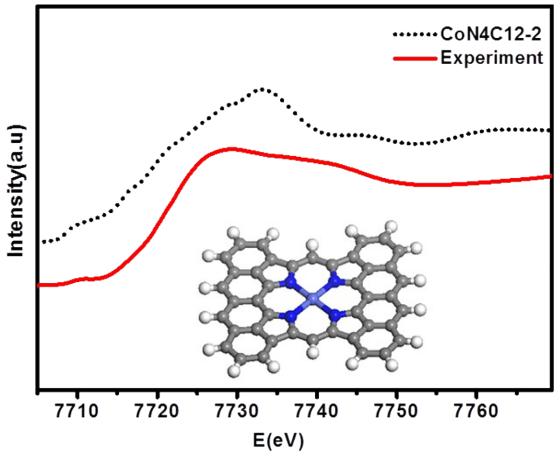
CoN4C12-2
Experiment
Intensity(a.u)
7710
7720
7730
7740
7750
7760
E(eV)

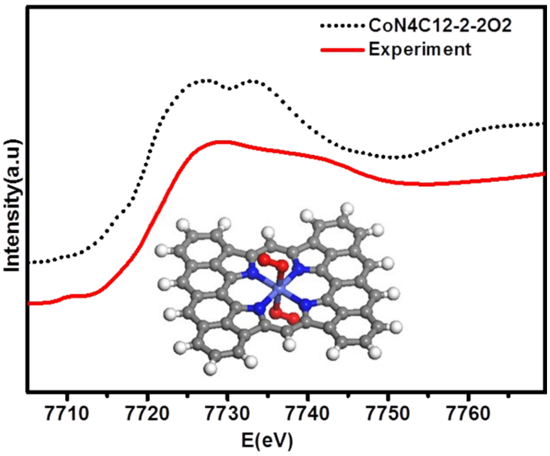
CoN4C12-2-2O2
Experiment
Intensity(a.u)
7710
7720
7730
7740
7750
7760
E(eV)

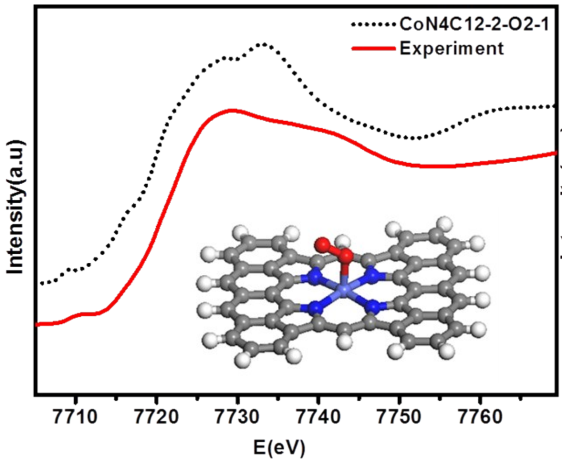
CoN4C12-2-O2-1
Experiment
Intensity(a.u)
7710
7720
7730
7740
7750
7760
E(eV)

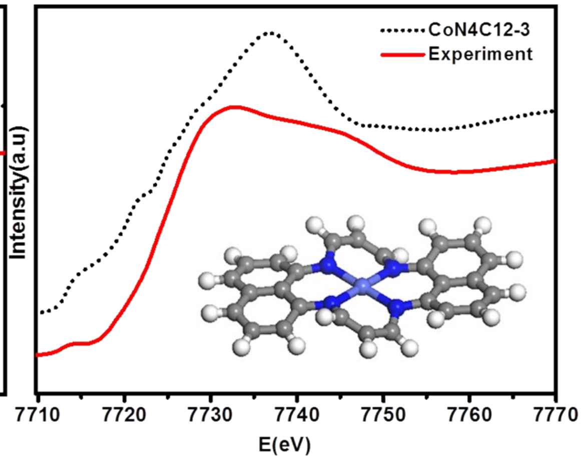
CoN4C12-3
Experiment
Intensity(a.u)
7710
7720
7730
7740
7750
7760
7770
E(eV)

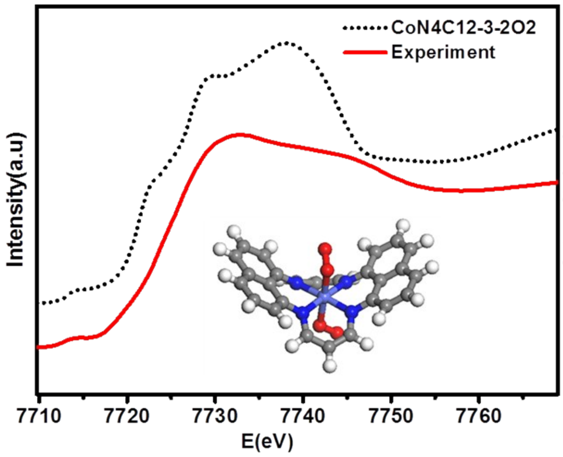
CoN4C12-3-2O2
Experiment
Intensity(a.u)
7710
7720
7730
7740
7750
7760
E(eV)

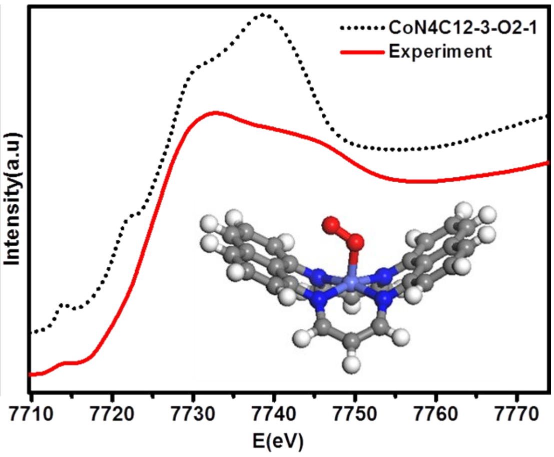
CoN4C12-3-O2-1
Experiment
Intensity(a.u)
7710
7720
7730
7740
7750
7760
7770
E(eV)

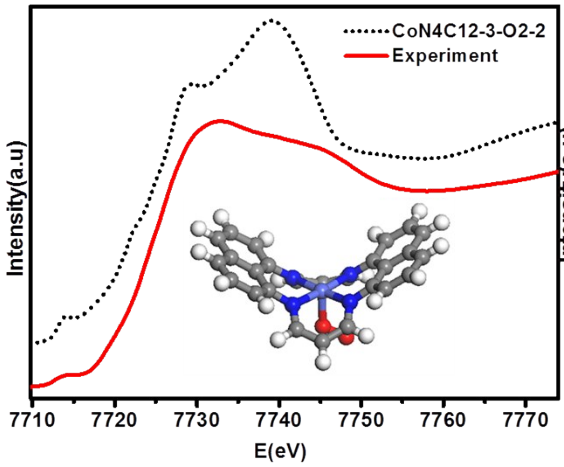
CoN4C12-3-O2-2
Experiment
Intensity(a.u)
7710
7720
7730
7740
7750
7760
7770
E(eV)

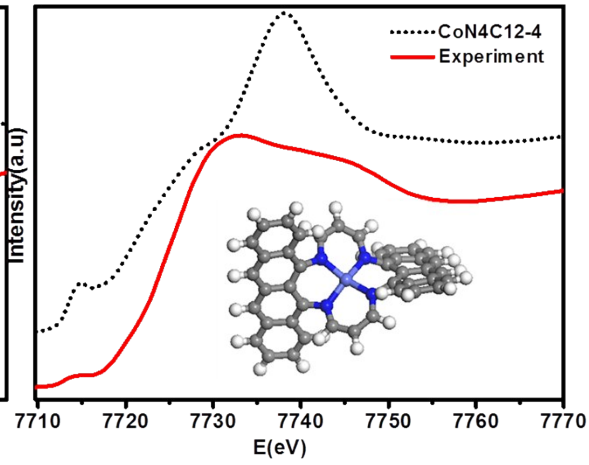
CoN4C12-4
Experiment
Intensity(a.u)
7710
7720
7730
7740
7750
7760
7770
E(eV)

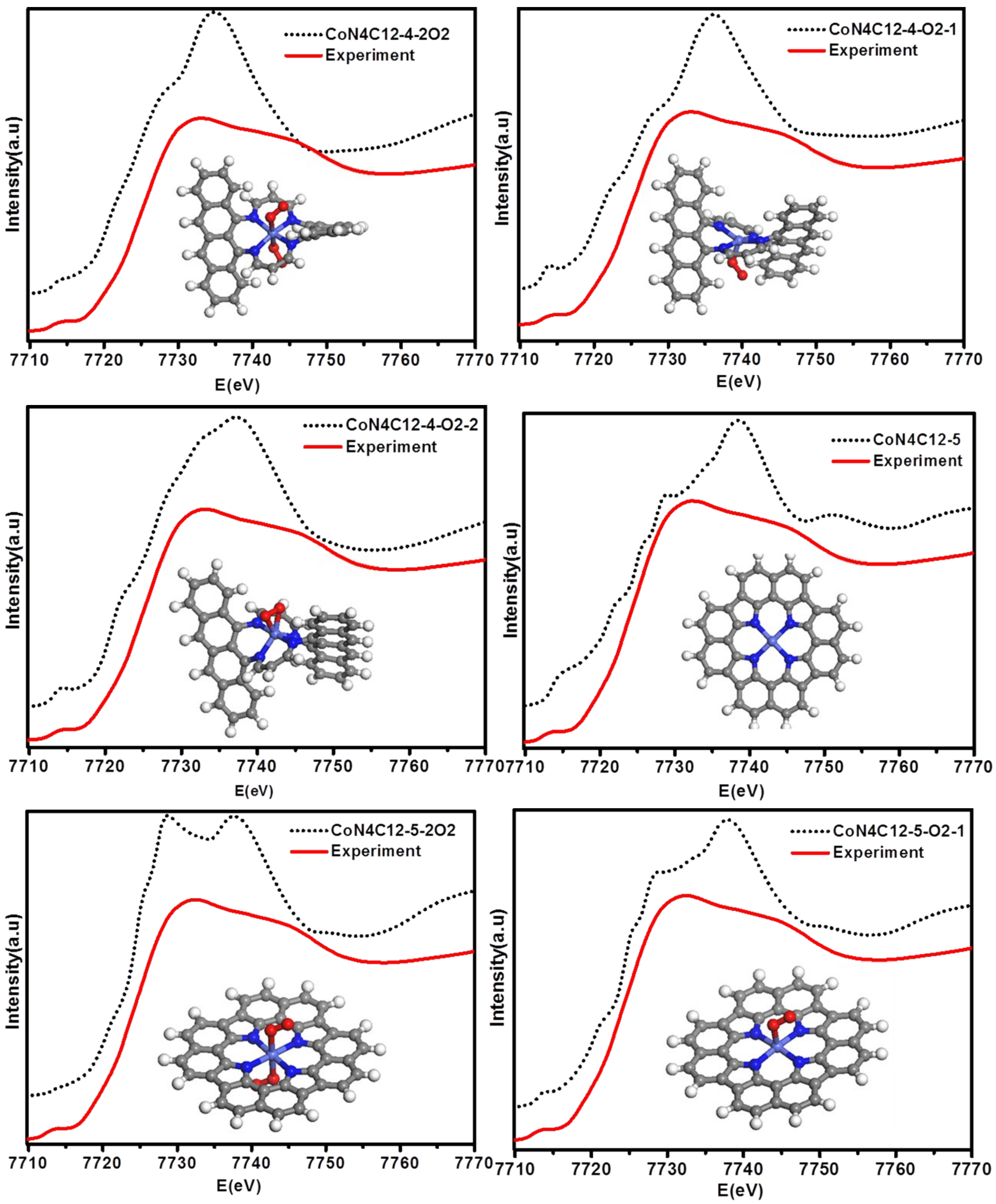
CoN4C12-4-2O2
Experiment
Intensity(a.u)
E(eV)
7710 7720 7730 7740 7750 7760 7770
CoN4C12-4-O2-1
Experiment
Intensity(a.u)
E(eV)
7710 7720 7730 7740 7750 7760 7770
CoN4C12-4-O2-2
Experiment
Intensity(a.u)
E(eV)
7710 7720 7730 7740 7750 7760 7770
CoN4C12-5
Experiment
Intensity(a.u)
E(eV)
7710 7720 7730 7740 7750 7760 7770
CoN4C12-5-2O2
Experiment
Intensity(a.u)
E(eV)
7710 7720 7730 7740 7750 7760 7770
CoN4C12-5-O2-1
Experiment
Intensity(a.u)
E(eV)
7710 7720 7730 7740 7750 7760 7770

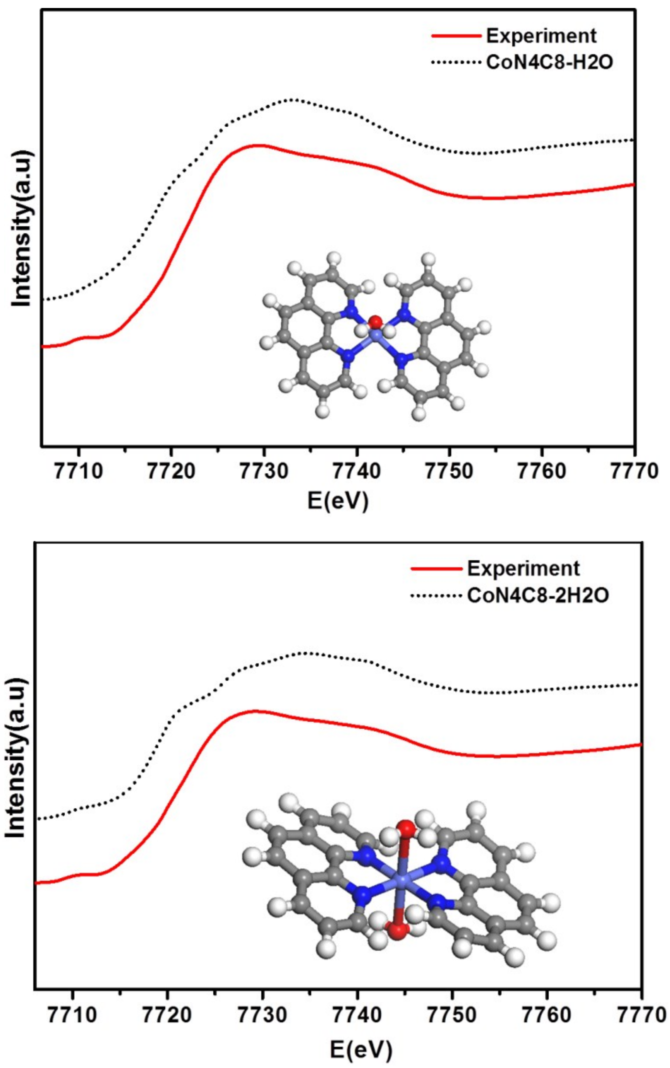

Figure S9. Comparison between the XANES experimental spectrum of Co-N-C at Co K edge (solid red lines) and the theoretical spectra calculated with the depicted structures (black dashed lines). In these calculations, the total scattering potentials including a fully relaxed core-hole were obtained iteratively, by successive calculations of the potential until self-consistency was reached. Based on this scattering potential, the final states of the excited photoelectron were then calculated. The Hedin-Lundqvist model of exchange potential with a 2 eV shift and additional broadening of 0.8 eV was used to give a closest match between the simulated and experimental spectra.

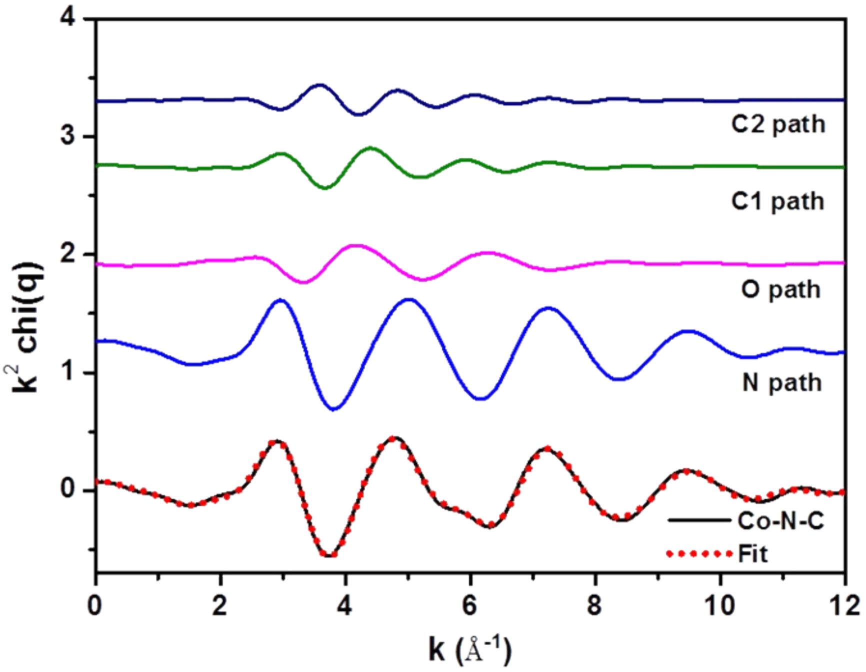

Figure S10. The contribution of every path including Co-N (blue line), Co-O (pink line) and Co-C (green and navy blue lines) in q-space for Co-N-C sample.

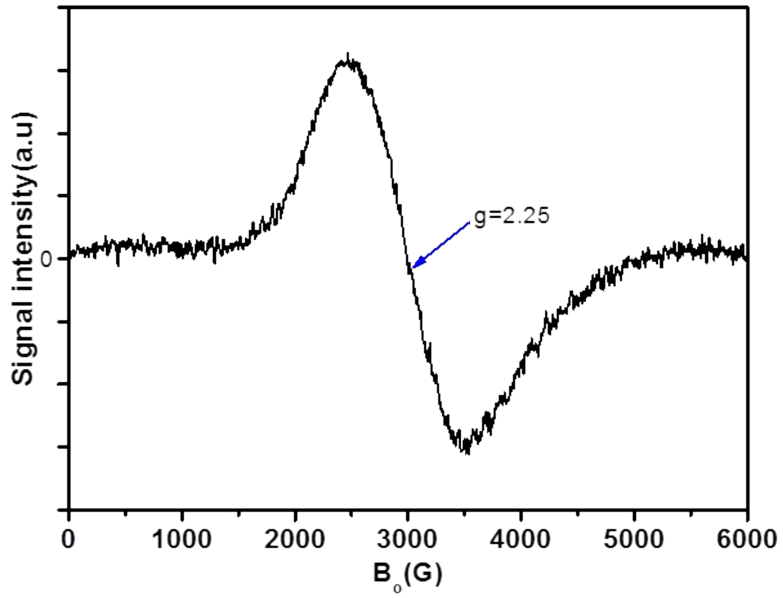

Figure S11. EPR spectra obtained at room temperature on the Co-N-C catalyst. A EPR signal with a Lorentzian shape and a g factor = 2.25 can be assigned to Co^{2+}.

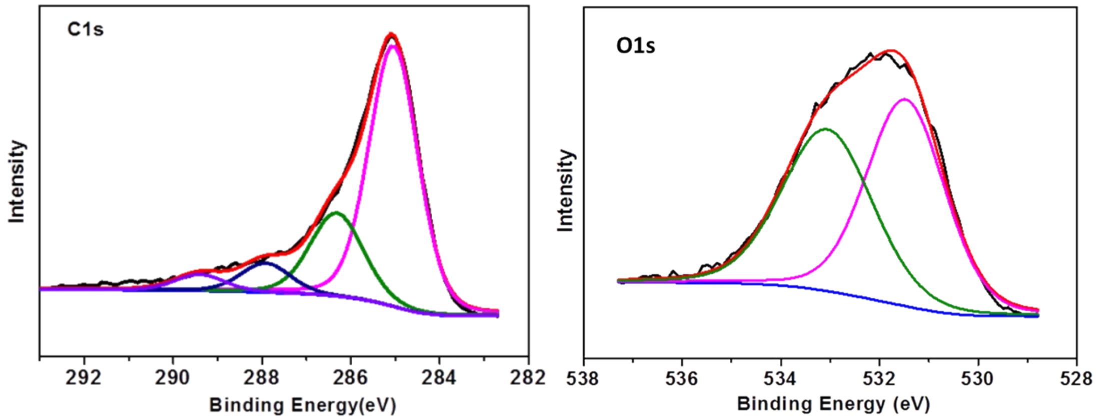

Figure S12. The C1s and O1s spectra of the Co-N-C catalyst. For the C1s spectrum, the four peaks with binding energies at 285, 286.4, 287.9 and 289.4 eV can be assigned to the graphitic C, C-O, C=O and C-OOH, respectively. For the O1s spectrum, the two peaks are ascribed to ketonic C=O groups (531.5 eV) and C−O groups (533.1 eV), respectively.

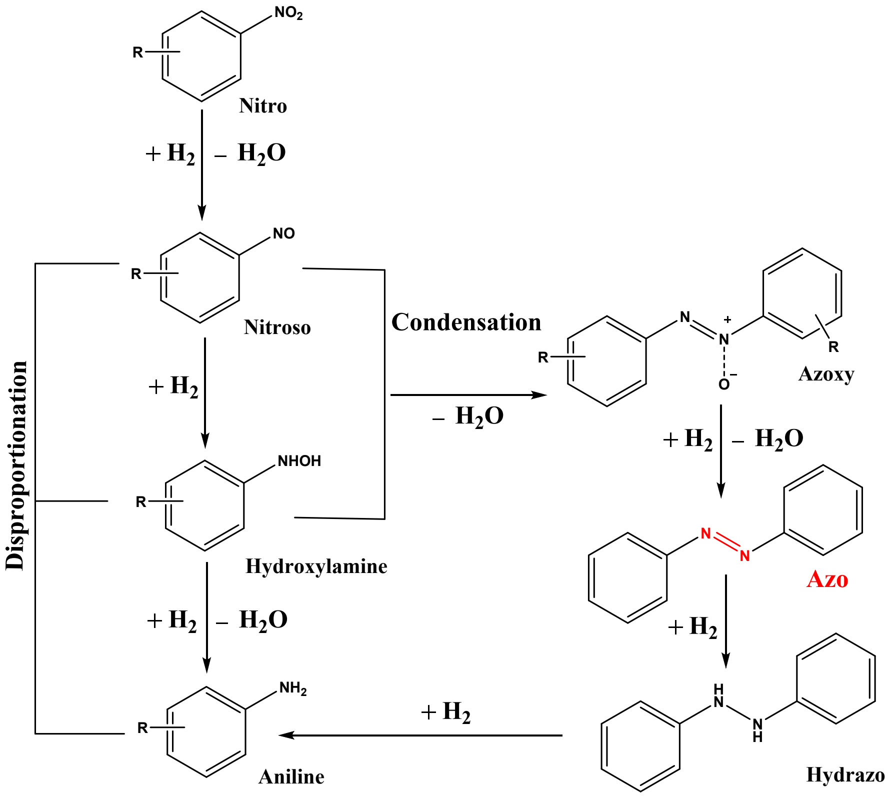

Figure S13. Proposed pathways for the reduction of nitroarenes on Co-N-C catalyst.

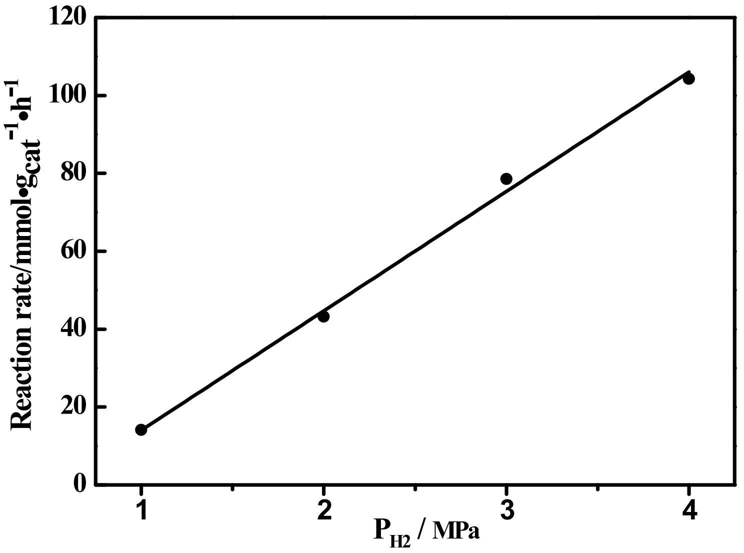

Figure S14. Dependency of the reaction rate on the pressure of H_2.

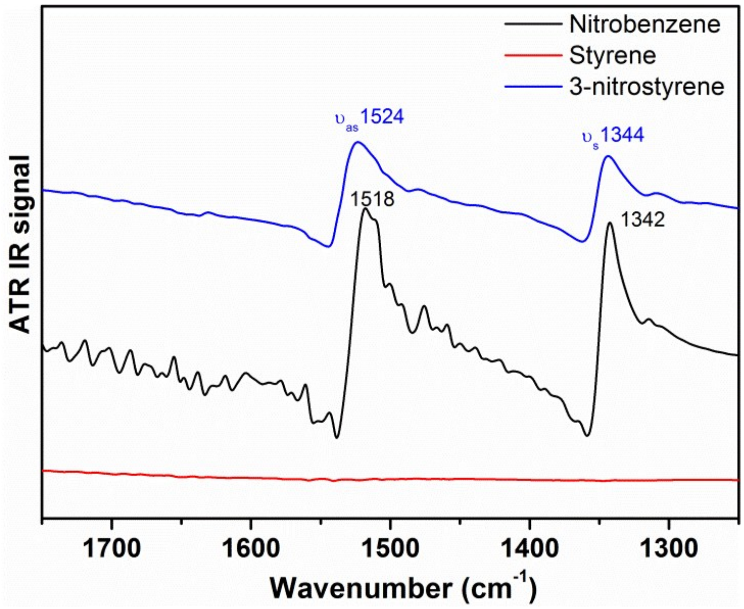

Figure S15. ATR-IR spectra of nitrobenzene, styrene and 3-nitrostyrene adsorbed on the Co-N-C catalyst at 25 ^{o}C.

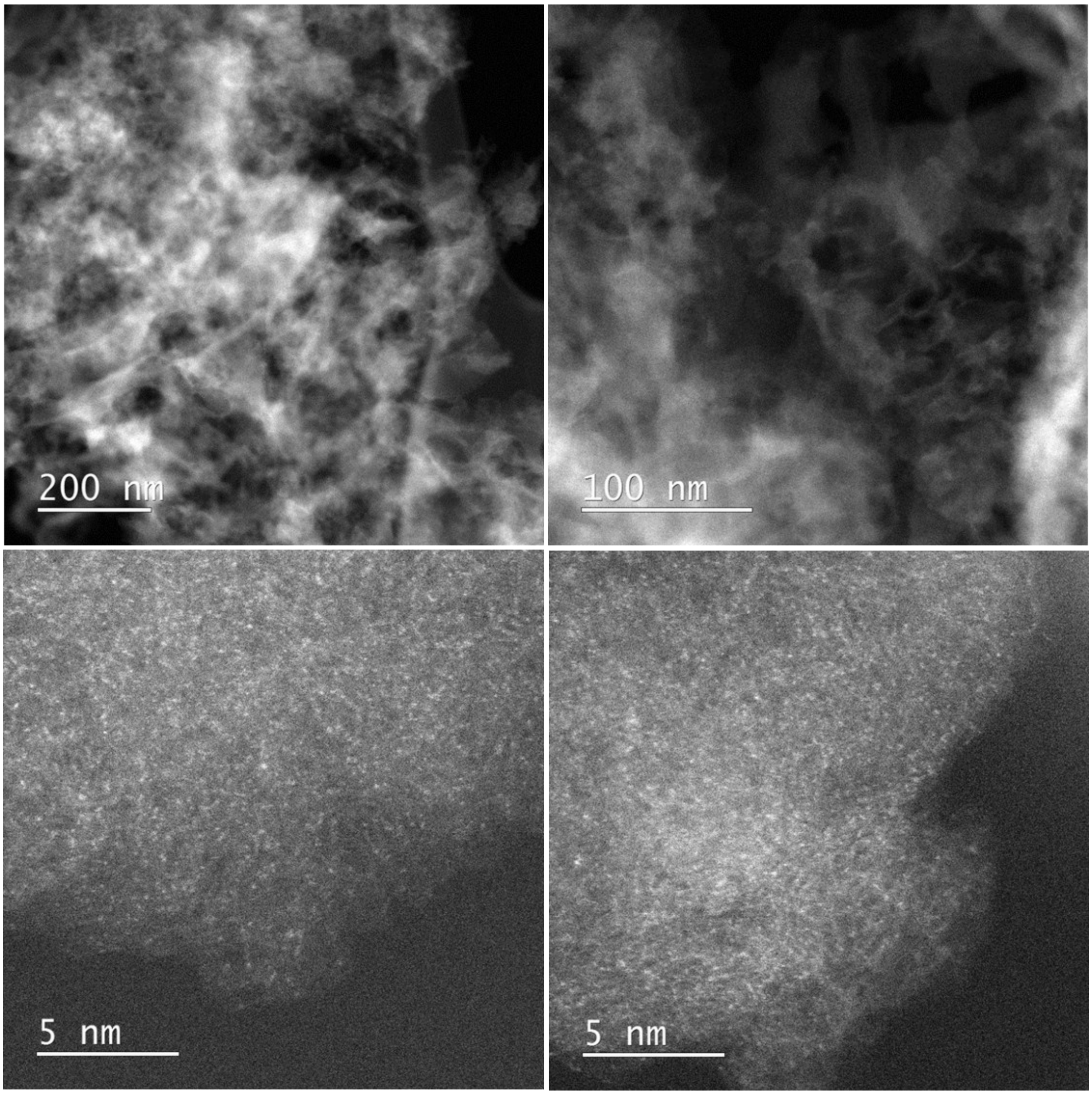

Figure S16. HAADF-STEM images of Co-N-C catalyst after reuse.

Table S1. E_0 value (the first inflection point) of the Co-N-C sample. CoPc = Cobalt phthalocyanine complex, CoPTT = Co porphyrin complex.

**Sample**	Co foil	Co-N-C -500	Co-N-C -700	Co-N-C -900	$Co(phen)_2(OAc)_2$	CoPc	CoPTT	Co_3O_4
**E_0(eV)**	7709	7719.2	7719.4	7709.2	7720.3	7714.8	7712.7	7727

Table S2. The optimized bond parameters of some selected models of Co-N-C, with the corresponding geometric structures.

	CoN_4C_8-1	CoN_4C_8-1-$2O_2$	CoN_4C_{10}-1	CoN_4C_{10}-1-$2O_2$

CoN1 / Å<br>CoN2 / Å<br>CoN3 / Å<br>CoN4 / Å	1.925<br>1.927<br>1.927<br>1.925	1.972<br>1.972<br>1.972<br>1.972	1.894<br>1.894<br>1.894<br>1.894	1.908<br>1.908<br>1.908<br>1.908
N1-Co-N2 /º<br>N3-Co-N4 /º<br>N1-Co-N4 /º<br>N2-Co-N3 /º	82.977<br>82.950<br>96.732<br>97.343	82.481<br>82.482<br>97.549<br>97.488	87.380<br>87.380<br>92.618<br>92.617	87.476<br>87.471<br>92.530<br>92.525
Co-O1 / Å<br>Co-O2 / Å		2.000<br>2.000		2.024<br>2.023
Co-C1 / Å<br>Co-C2 / Å	~2.76<br>~3.02	~2.78<br>~3.04	~2.74<br>~2.96	~2.70<br>~2.96

	CoN_4C_{12}-3	CoN_4C_{12}-3-$2O_2$	CoN_4C_{12}-4	CoN_4C_{12}-4-$2O_2$
CoN1 / Å<br>CoN2 / Å<br>CoN3 / Å<br>CoN4 / Å	1.990<br>1.981<br>1.990<br>1.982	1.954<br>1.946<br>1.950<br>1.956	1.899<br>1.915<br>1.900<br>1.915	1.991<br>1.999<br>1.984<br>1.992
N1-Co-N2 /º<br>N3-Co-N4 /º<br>N1-Co-N4 /º<br>N2-Co-N3 /º	87.008<br>87.037<br>92.990<br>92.965	88.172<br>88.397<br>91.634<br>91.547	92.193<br>92.088<br>94.907<br>94.842	88.829<br>88.741<br>93.722<br>93.667
Co-O1 / Å<br>Co-O2 / Å		1.972<br>2.058		1.991<br>2.035

Table S3. Surface composition of Co-N-C catalyst determined by XPS.

Entry	Elements	Atomic (%)
1	C 1s	79.26
2	N 1s	8.66
3	O 1s	11.36
4	Co 2p	0.66
5	Mg 1s	0.06

2. Optimization of the reaction conditions, and control experiment results

Table S4. Optimization of the reaction conditions.

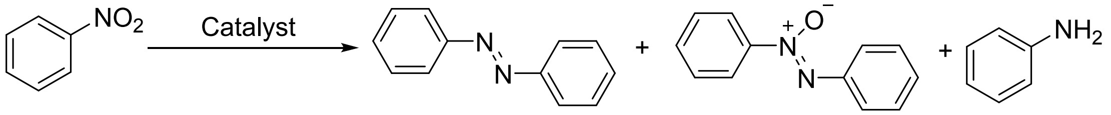

Entry	Solvent	T(°C)	P(MPa)	Base	Conv.(%)	Yield(%)		
						2a	2b	2c
1	Toluene	80	3	NaOH	47	0	33	7
2	THF	80	3	NaOH	100	7	89	0
3	Ethanol	80	3	NaOH	83	0	39	14
4	TBA	80	2	NaOH	100	17	82	0
5	TBA	80	1	NaOH	46	0	46	0
6	TBA	60	3	NaOH	58	0	58	0
7	TBA	40	3	NaOH	30	0	28	0
8	TBA	80	3	Na_2CO_3	14	0	0	5
9	TBA	80	3	$NaHCO_3$	15	0	0	10
10	TBA	80	3	NaOH	100	99	0	0

Reaction conditions：3.6 wt% Co-N-C catalyst (0.7 mol%Co), 1.0 mmol nitrobenzene，Base 0.2 mmol, H_2，2 mL solvent，1.5 h. Yield were Determined by GC, using 100 μl Dodecane as an internal standard. TBA=tert butyl alcohol.

Table S5. Hydrogenation of nitrobenzene and styrene over Co-N-C based catalysts.

Feed (mmol)		TOF (h^{-1})	
nitrobenzene	styrene	nitrobenzene	styrene
1	0	271	-
0	1	-	0
0.5	0.5	268	0

Reation conditions：3.6 wt% Co-N-C catalyst (0.7 mol%Co), substrate，NaOH 0.2 mmol, 3 MPa H_2，80℃，20 min, 2 mL TBA solvent, TBA=tert butyl alcohol.

Table S6. Recovery and reuse of Co-N-C based catalyst.

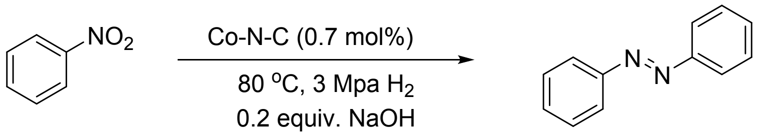

Recycle	1st	2nd	3rd	4th	5th
Reaction time (h)	1.5	2	2.5	3	3.5
Conversion (%)	100	100	100	100	100
Yield (%)	99	94	99	100	98

3. Characterization of the obtained products

All chemicals (Analytical Grade) were used as received without further purification. The aerobic oxidative coupling reactions were monitored with analytical thin-layer chromatography (TLC) on silica gel 60 F_{254} plates and visualized under UV (254 nm).

Gas chromatography (GC) analysis was performed on an Agilent 7890B system equipped with a 5% phenyl methyl siloxane capillary column (30 m × 320 μm × 0.25 μm). The GC yield was obtained from the calibration curve using dodecane as an internal standard.

^{1}H NMR spectra were recorded on commercial instruments (500 MHz). Chemical shifts were reported in ppm from tetramethylsilane with the solvent resonance as the internal standard ($CDCl_3$, δ = 7.26). ^{13}C NMR spectra were collected on commercial instruments (125 MHz) with complete proton decoupling. Chemical shifts are reported in ppm from the tetramethylsilane with the solvent resonance as internal standard ($CDCl_3$, δ = 77.0). The structures of the known compounds were confirmed by comparison with commercially available compounds or data published in literature.

The structures of the known compounds were confirmed by comparison with commercially available compounds or data published in literature.

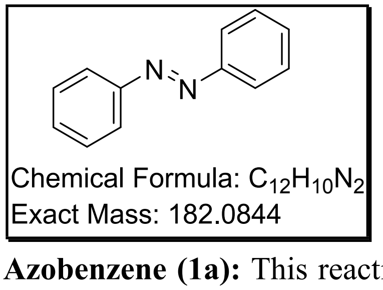

Azobenzene (1a): This reaction was carried out according to general procedure with nitrobenzene(102 μL, 1 mmol, 1 equiv). The crude reaction mixture was purified on silica gel (2% ethyl acetate/petroleum ether) to afford the product as a yellow red solid (97% yield). ^{1}H NMR (500 MHz, CDC_{l3}) δ 7.95 – 7.90 (m, J = 7.3 Hz, 4H), 7.54 – 7.46 (m, 6H).^{13}C NMR (126 MHz, CDC_{l3}) δ 152.67, 130.92, 129.03, 122.81.

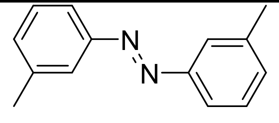

Chemical Formula: $C_{14}H_{14}N_2$
Exact Mass: 210.1157

3,3'-Dimethylazobenzene(2a): This reaction was carried out according to general procedure with 3-nitrotoluene (119 µL, 1 mmol, 1 equiv). The crude reaction mixture was purified on silica gel (2% ethyl acetate/petroleum ether) to afford the product as a yellow red solid (94% yield). ^{1}H NMR (500 MHz, $CDCl_3$) δ 7.98 (d, J = 6.5 Hz, 4H), 7.67 – 7.63 (m, 2H), 7.53 (d, J = 7.5 Hz, 2H), 2.70 (s, 6H). ^{13}C NMR (126 MHz, $CDCl_3$) δ 152.81, 138.94, 131.68, 128.88, 122.89, 120.46, 21.36.

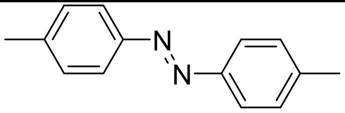

Chemical Formula: $C_{14}H_{14}N_2$
Exact Mass: 210.1157

4,4'-Dimethylazobenzene(3a): This reaction was carried out according to general procedure with 4-nitrotoluene (137.1 mg, 1 mmol, 1 equiv). The crude reaction mixture was purified on silica gel (2% ethyl acetate/petroleum ether) to afford the product as a yellow solid (88% yield). ^{1}H NMR (500 MHz, $CDCl_3$) δ 8.05 (d, J = 8.3 Hz, 4H), 7.54 (d, J = 8.1 Hz, 4H), 2.66 (s, 6H). ^{13}C NMR (126 MHz, $CDCl_3$) δ 150.83, 141.17, 129.68, 122.71, 21.45.

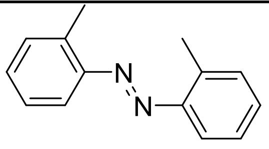

Chemical Formula: $C_{14}H_{14}N_2$
Exact Mass: 210.1157

2,2'-Dimethylazobenzene(4a): This reaction was carried out according to general procedure with 2-nitrotoluene (118 µL, 1 mmol, 1 equiv). The crude reaction mixture was purified on silica gel (2% ethyl acetate/petroleum ether) to afford the product as a red solid (63% yield). ^{1}H NMR (500 MHz, $CDCl_3$) δ 7.62 (d, J = 7.9 Hz, 2H), 7.37 – 7.32 (m, 4H), 7.28 – 7.24 (m, 2H), 2.74 (s, 6H); ^{13}C NMR (126 MHz, $CDCl_3$) δ 151.11, 137.93, 131.21, 130.61, 126.31, 115.84, 17.54.

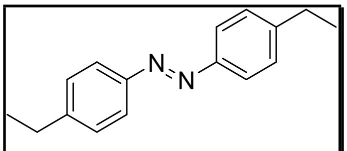

Chemical Formula: $C_{16}H_{18}N_2$
Exact Mass: 238.1470

2,2'-Dimethylazobenzene(5a): This reaction was carried out according to general procedure with 4-nitroethylbenzene (135 μL, 1 mmol, 1 equiv). The crude reaction mixture was purified on silica gel (2% ethyl acetate/petroleum ether) to afford the product as a yellow solid (85% yield). ^{1}H NMR (400 MHz, $CDCl_3$) δ 7.87 – 7.81 (m, 4H), 7.33 (d, J = 7.4 Hz, 4H), 2.72 (q, J = 7.5 Hz, 4H), 1.29 (ddd, J = 7.6, 4.3, 1.1 Hz, 6H). ^{13}C NMR (101 MHz, $CDCl_3$) δ 157.46, 151.10, 147.45, 128.51, 122.83, 28.84, 15.41.

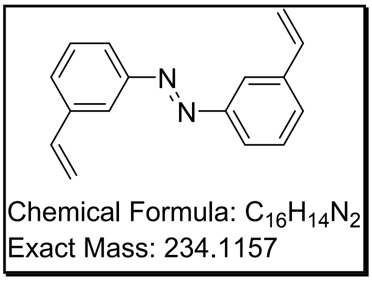

1,2-Bis(3-vinylphenyl)diazene (6a): This reaction was carried out according to general procedure with 3-nitrostyrene (145 μL, 1 mmol, 1 equiv). The crude reaction mixture was purified on silica gel (2% ethyl acetate/petroleum ether) to afford the product as a yellow solid (99% yield). ^{1}H NMR (400 MHz, $CDCl_3$) δ 7.89 (t, J = 1.7 Hz, 2H), 7.74 (dt, J = 7.6, 1.6 Hz, 2H), 7.46 – 7.35 (m, 4H), 6.73 (dd, J = 17.6, 10.9 Hz, 2H), 5.82 – 5.77 (m, 2H), 5.27 (d, J = 10.9 Hz, 2H).^{13}C NMR (101 MHz, $CDCl_3$) δ 152.94, 138.71, 136.24, 129.23, 128.78, 122.17, 120.62, 115.05.

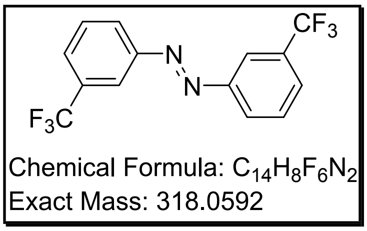

1,2-Bis(3-(trifluoromethyl)phenyl)diazene(7a): This reaction was carried out according to general procedure with 3-nitrobenzotrifluoride (137 μL, 1 mmol, 1 equiv). The crude reaction mixture was purified on silica gel (2% ethyl acetate/petroleum ether) to afford the product as a red solid (85% yield). ^{1}H NMR (500 MHz, $CDCl_3$) δ 8.22 (s, 2H), 8.14 (d, J = 7.9 Hz, 2H), 7.78 (d, J = 7.7 Hz, 2H), 7.68 (t, J = 7.8 Hz, 2H).^{13}C NMR (126 MHz, $CDCl_3$) δ 152.18, 131.99, 131.72, 129.79, 127.84 (d, J = 3.6 Hz, 1H), 126.45, 124.80, 122.64, 119.72 (q, J = 3.7 Hz, 1H).

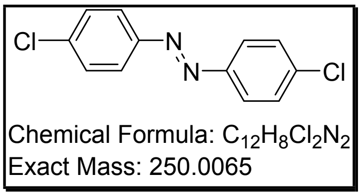

1,2-Bis(4-chlorophenyl)diazene (8a): This reaction was carried out according to general procedure with 4-chloronitrobenzene (158 mg, 1 mmol, 1 equiv). The crude

reaction mixture was purified on silica gel (2% ethyl acetate/petroleum ether) to afford the product as a yellow solid (97% yield). ^{1}H NMR (500 MHz, $CDCl_3$) δ 7.86 (d, J = 8.0 Hz, 4H), 7.49 (d, J = 7.9 Hz, 4H). ^{13}C NMR (126 MHz, $CDCl_3$) δ 150.79, 137.20, 129.36, 124.15.

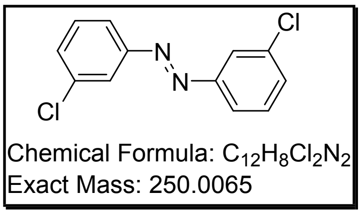

1,2-Bis(3-chlorophenyl)diazene (9a): This reaction was carried out according to general procedure with 3-chloronitrobenzene (158 mg, 1 mmol, 1 equiv). The crude reaction mixture was purified on silica gel (2% ethyl acetate/petroleum ether) to afford the product as a yellow solid (98% yield). ^{1}H NMR (500 MHz, $CDCl_3$) δ 7.89 (s, 2H), 7.83 (d, J = 4.0 Hz, 2H), 7.46 (d, J = 3.9 Hz, 4H). ^{13}C NMR (126 MHz, $CDCl_3$) δ 153.11, 135.22, 131.16, 130.15, 122.55, 121.89.

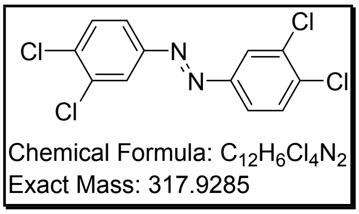

1,2-Bis(3,4-dichlorophenyl)diazene(10a): This reaction was carried out according to general procedure with 3,4-dichloronitrobenzene (202.1 mg, 1 mmol, 1 equiv). The crude reaction mixture was purified on silica gel (2% ethyl acetate/petroleum ether) to afford the product as a yellow solid (99% yield). ^{1}H NMR (500 MHz, $CDCl_3$) δ 8.02 (s, 2H), 7.80 (d, J = 8.5 Hz, 2H), 7.62 (d, J = 8.5 Hz, 2H). ^{13}C NMR (126 MHz, $CDCl_3$) δ 151.02, 135.68, 133.66, 130.99, 124.05, 123.01.

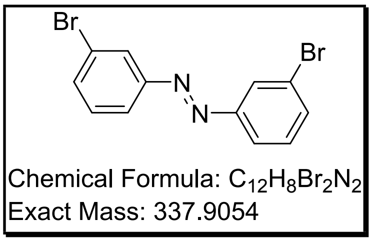

1,2-Bis(3-bromophenyl)diazene(11a): This reaction was carried out according to general procedure with 1-bromo-3-nitrobenzene (206.1 mg, 1 mmol, 1 equiv). The crude reaction mixture was purified on silica gel (2% ethyl acetate/petroleum ether) to afford the product as a yellow solid (96% yield). ^{1}H NMR (500 MHz, $CDCl_3$) δ 8.05 (s, 1H), 7.88 (d, J = 7.7 Hz, 1H), 7.62 (d, J = 7.7 Hz, 1H), 7.41 (t, J = 7.9 Hz, 1H). ^{13}C NMR (126 MHz, $CDCl_3$) δ 153.19, 134.06, 130.44, 124.77, 123.15, 123.07.

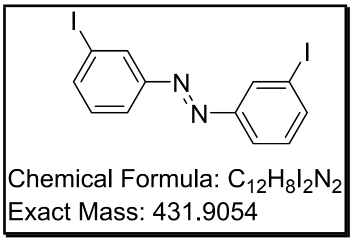

1,2-Bis(3-iodophenyl)diazene(12a): This reaction was carried out according to general procedure with 3-iodonitrobenzene (249.1 mg, 1 mmol, 1 equiv). The crude reaction mixture was purified on silica gel (2% ethyl acetate/petroleum ether) to afford the product as a yellow solid (94% yield). ^{1}H NMR (500 MHz, $CDCl_3$) δ 8.24 (s, 2H), 7.90 (d, J = 7.8 Hz, 2H), 7.81 (d, J = 7.7 Hz, 2H), 7.31 – 7.26 (m, 2H). ^{13}C NMR (126 MHz, $CDCl_3$) δ 153.06, 139.96, 130.77, 130.64, 123.63, 94.48.

4. Copy of ^{1}H NMR and ^{13}C NMR spectra for products

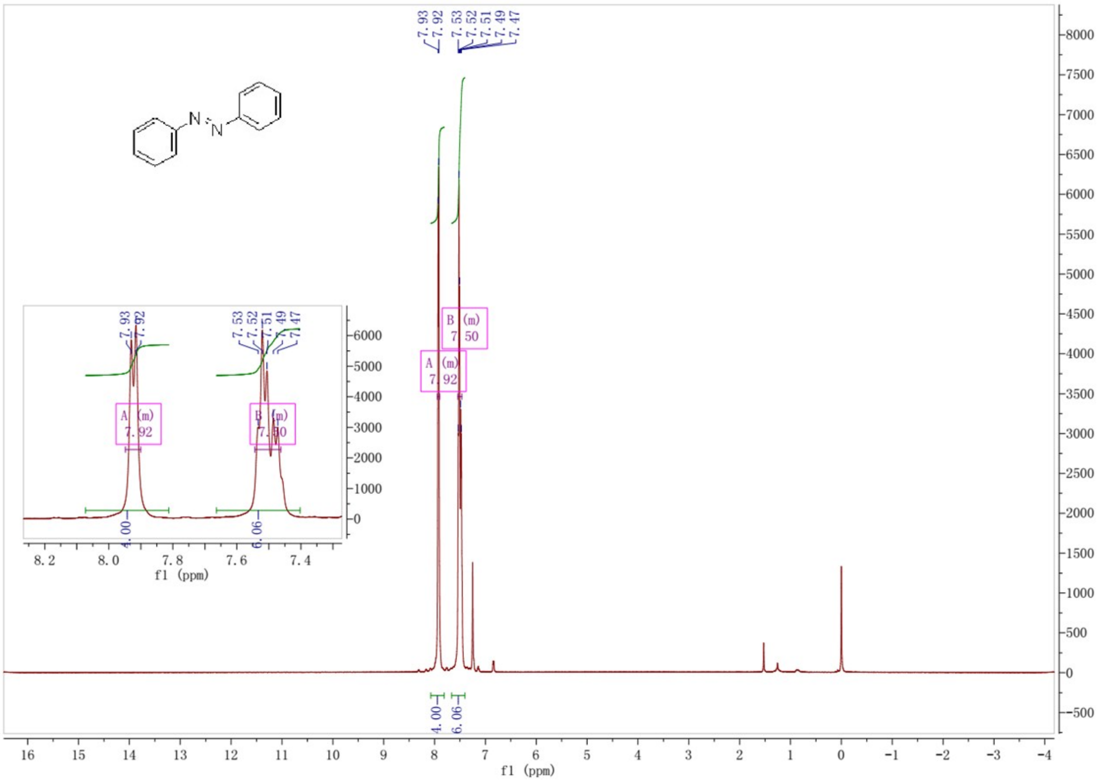

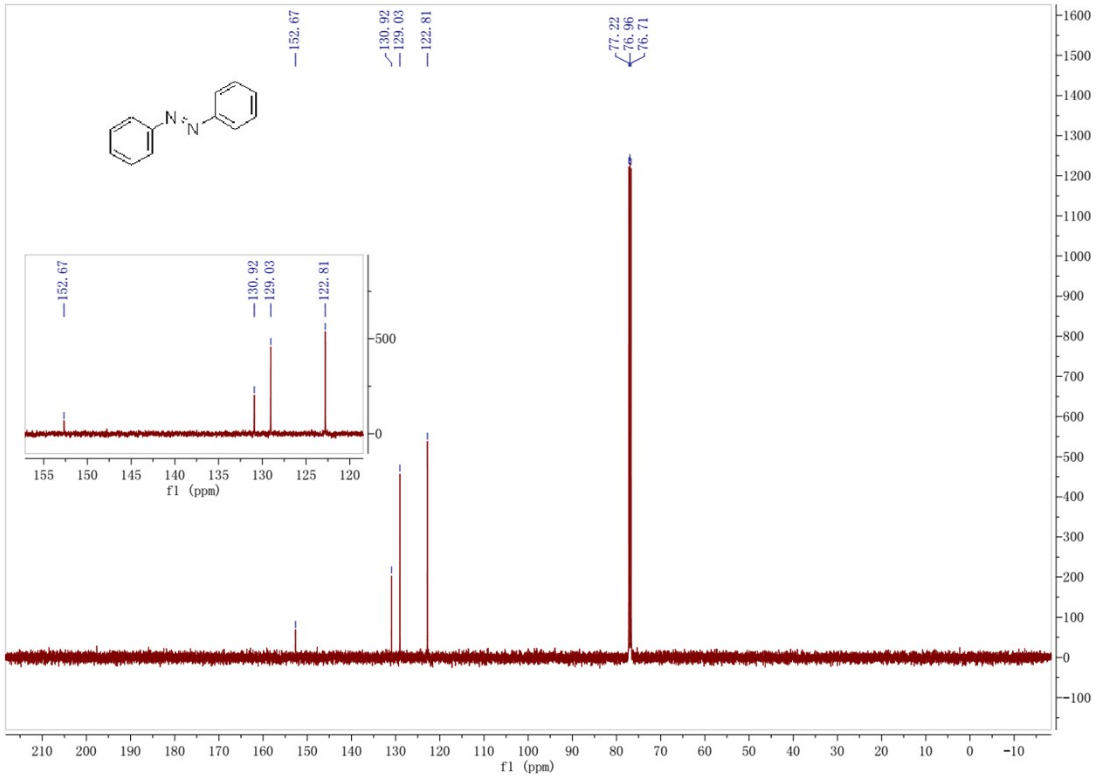

152.67
130.92
129.03
122.81
77.22
76.96
76.71
f1 (ppm)

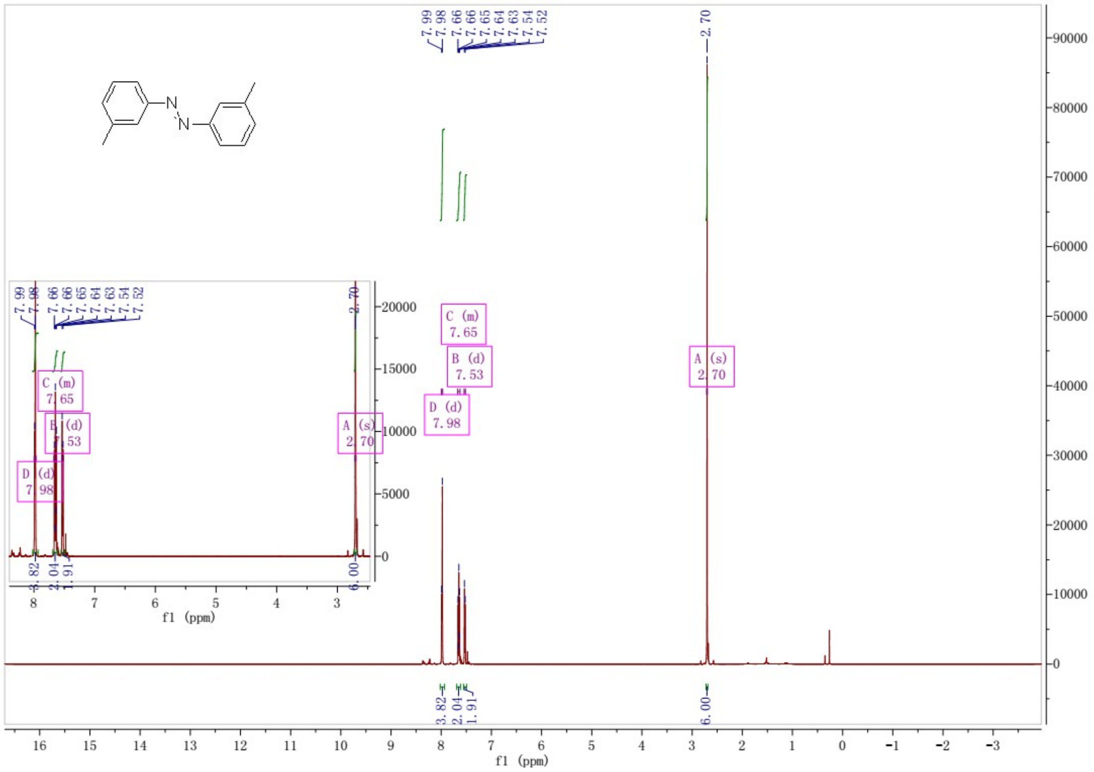

7.99
7.98
7.66
7.66
7.65
7.64
7.63
7.54
7.52
2.70
C (m) 7.65
B (d) 7.53
D (d) 7.98
A (s) 2.70
3.82
2.04
1.91
6.00
f1 (ppm)

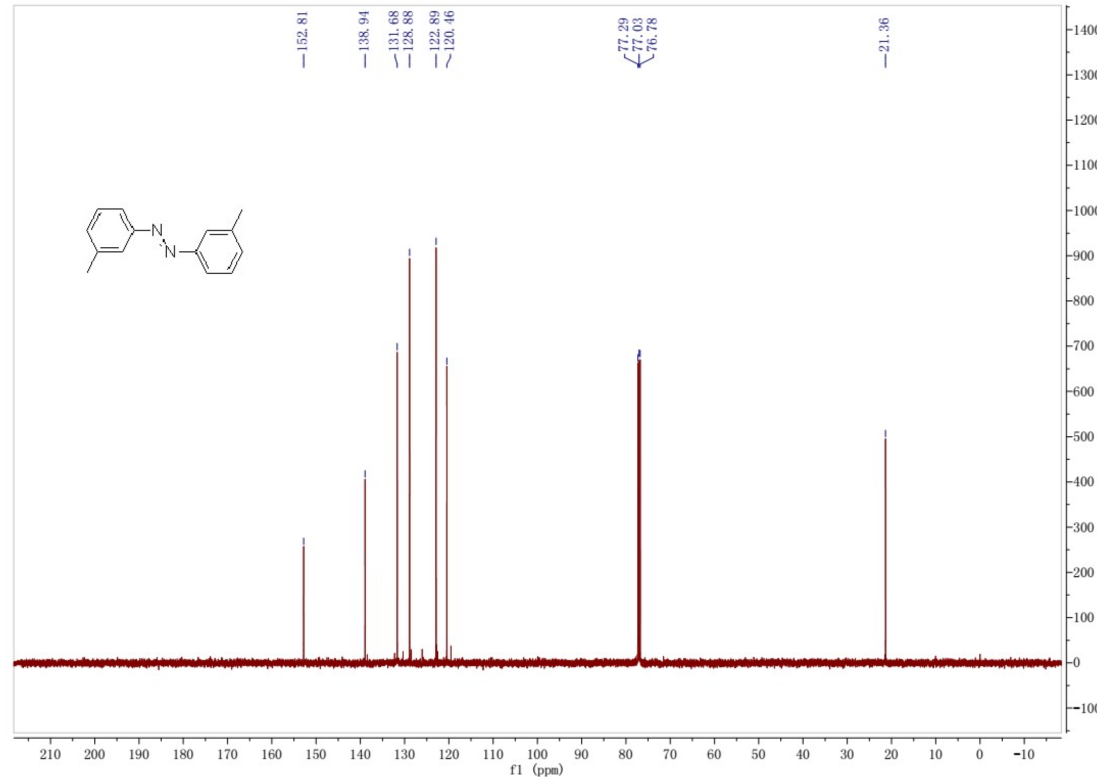
152.81
138.94
131.68
128.88
122.89
120.46
77.29
77.03
76.78
21.36
f1 (ppm)

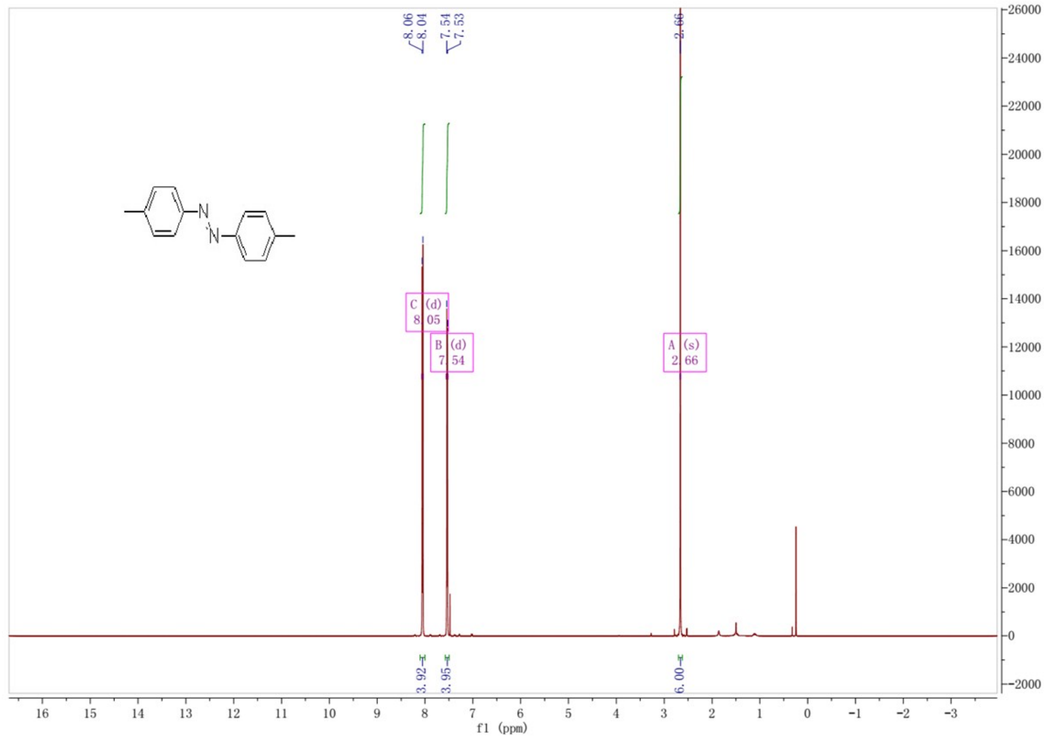
8.06
8.04
7.54
7.53
2.66
C (d)
8.05
B (d)
7.54
A (s)
2.66
3.92
3.95
6.00
f1 (ppm)

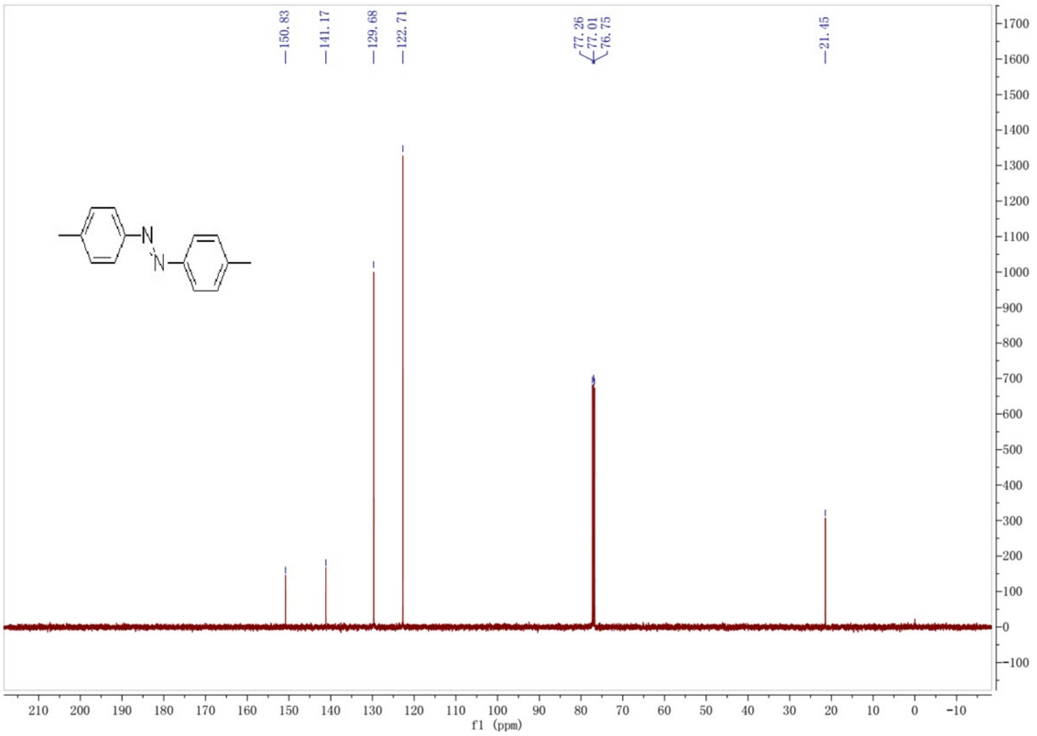
150.83
141.17
129.68
122.71
77.26
77.01
76.75
21.45
f1 (ppm)

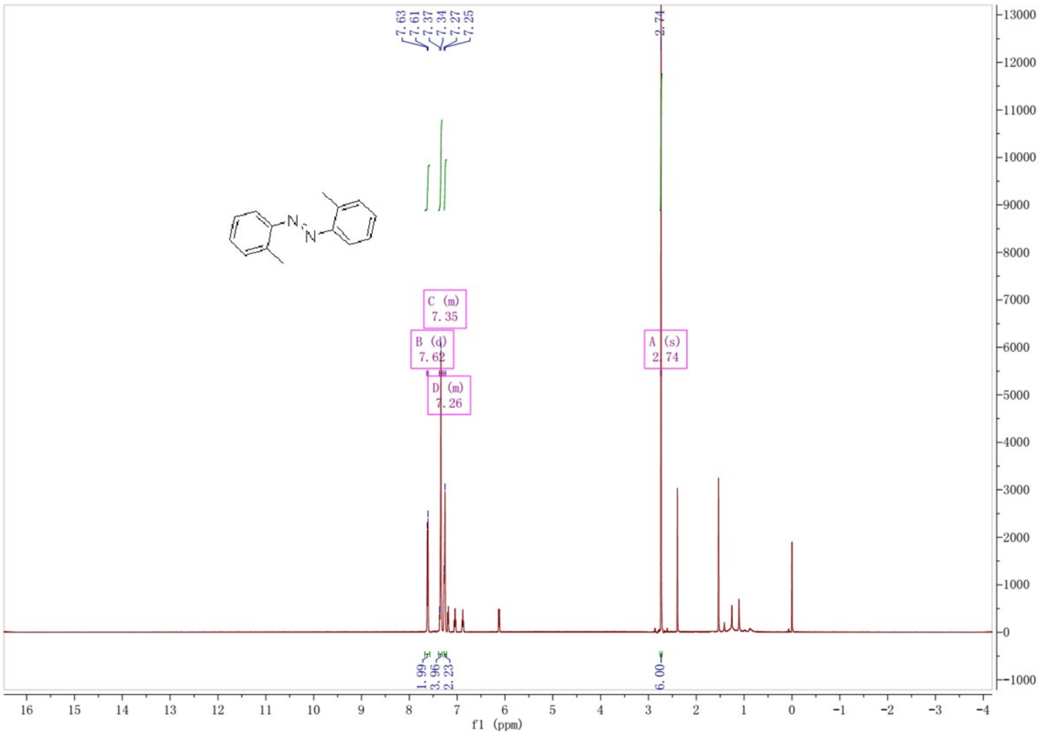
7.63
7.61
7.37
7.34
7.27
7.25
2.74
C (m)
7.35
B (d)
7.62
D (m)
7.26
A (s)
2.74
1.99
3.96
2.23
6.00
f1 (ppm)

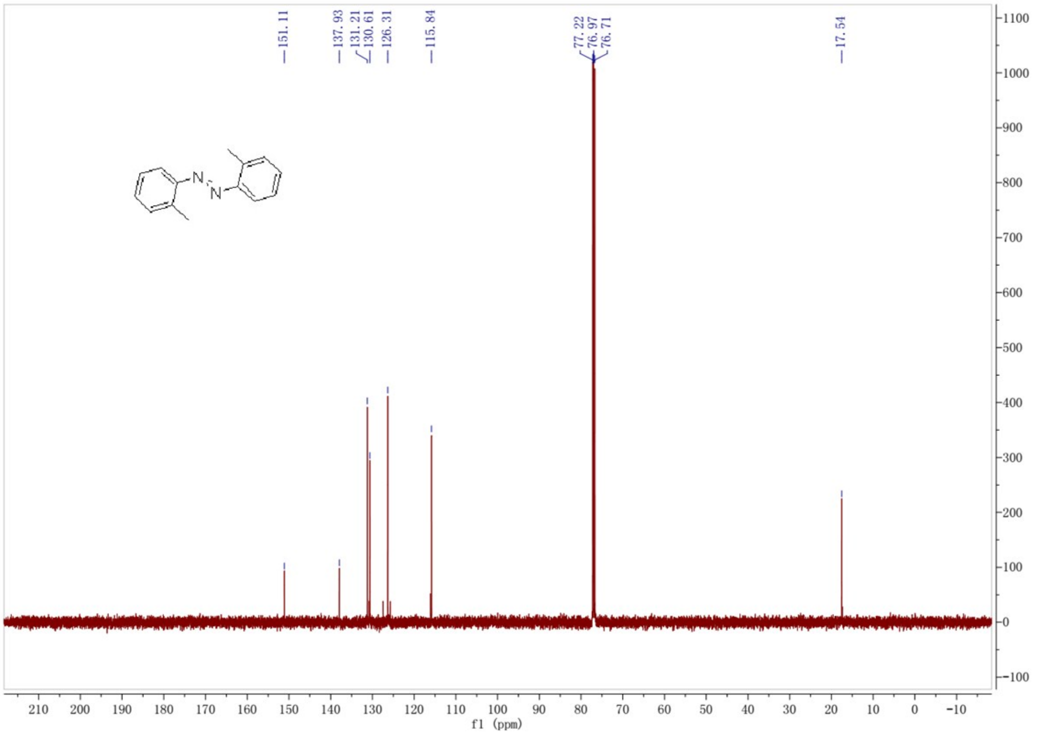
151.11
137.93
131.21
130.61
126.31
115.84
77.22
76.97
76.71
17.54
f1 (ppm)

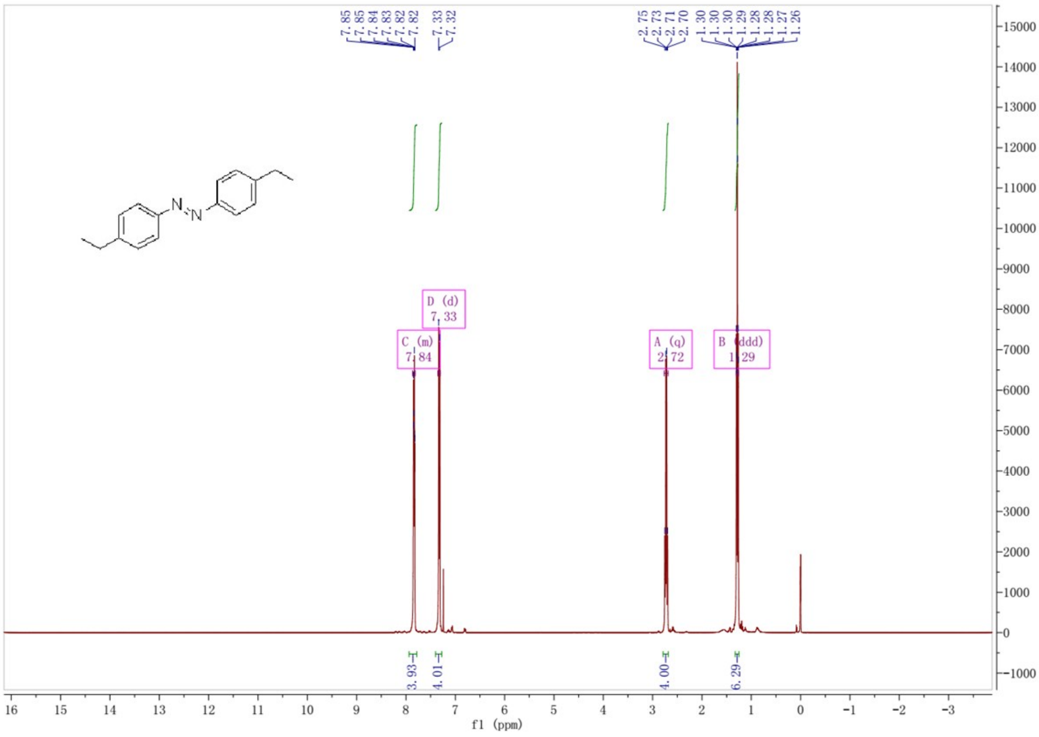
7.85
7.85
7.84
7.83
7.82
7.82
7.82
7.33
7.32
2.75
2.73
2.71
2.70
1.30
1.30
1.30
1.29
1.28
1.28
1.27
1.26
D (d)
7.33
C (m)
7.84
A (q)
2.72
B (ddd)
1.29
3.93
4.01
4.00
6.29
f1 (ppm)

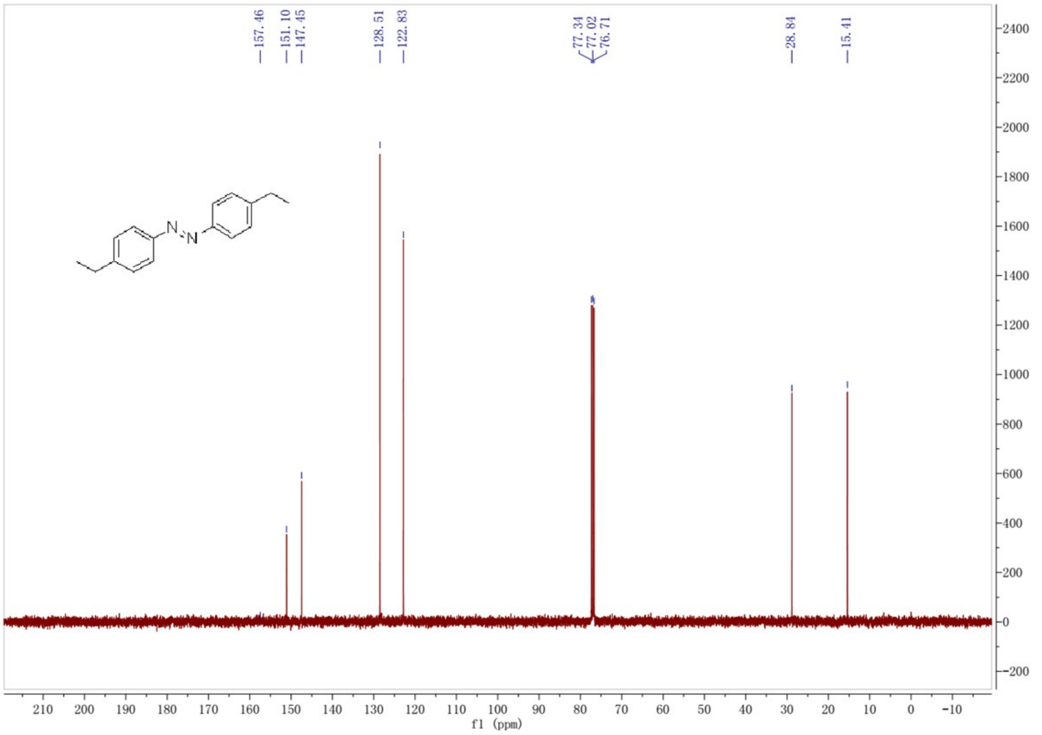
157.46
151.10
147.45
128.51
122.83
77.34
77.02
76.71
28.84
15.41
f1 (ppm)

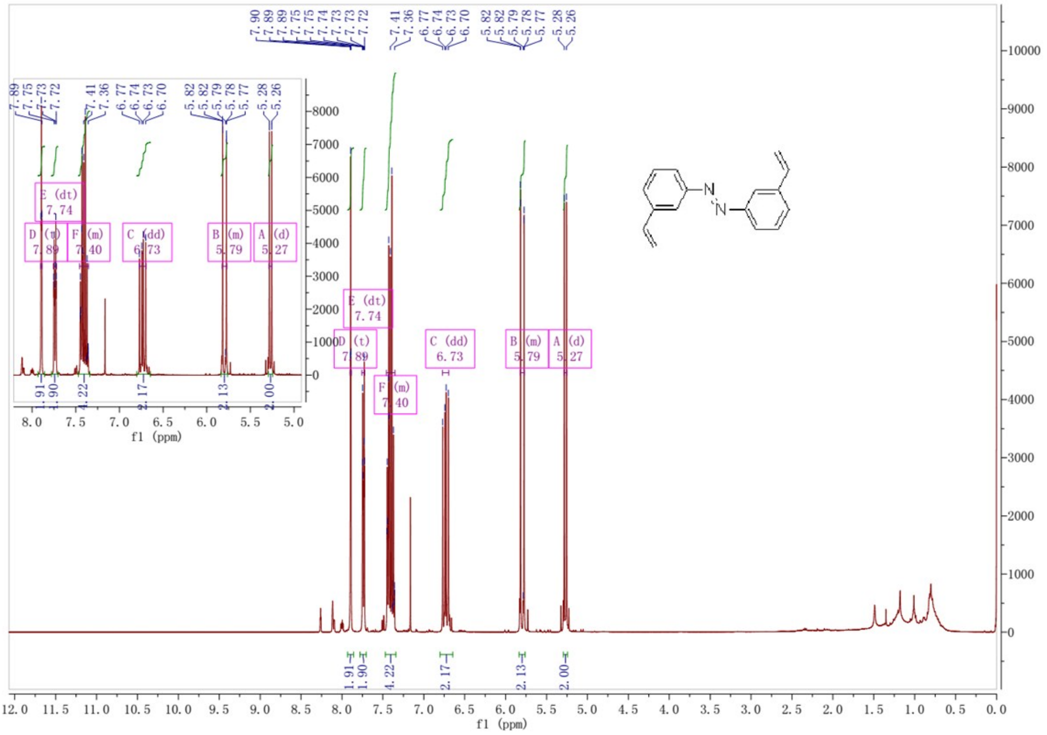
7.90
7.89
7.89
7.75
7.75
7.74
7.73
7.73
7.72
7.41
7.36
6.77
6.74
6.73
6.70
5.82
5.82
5.79
5.78
5.77
5.28
5.26
D (t) 7.89
E (dt) 7.74
F (m) 7.40
C (dd) 6.73
B (m) 5.79
A (d) 5.27
1.91
1.90
4.22
2.17
2.13
2.00
f1 (ppm)

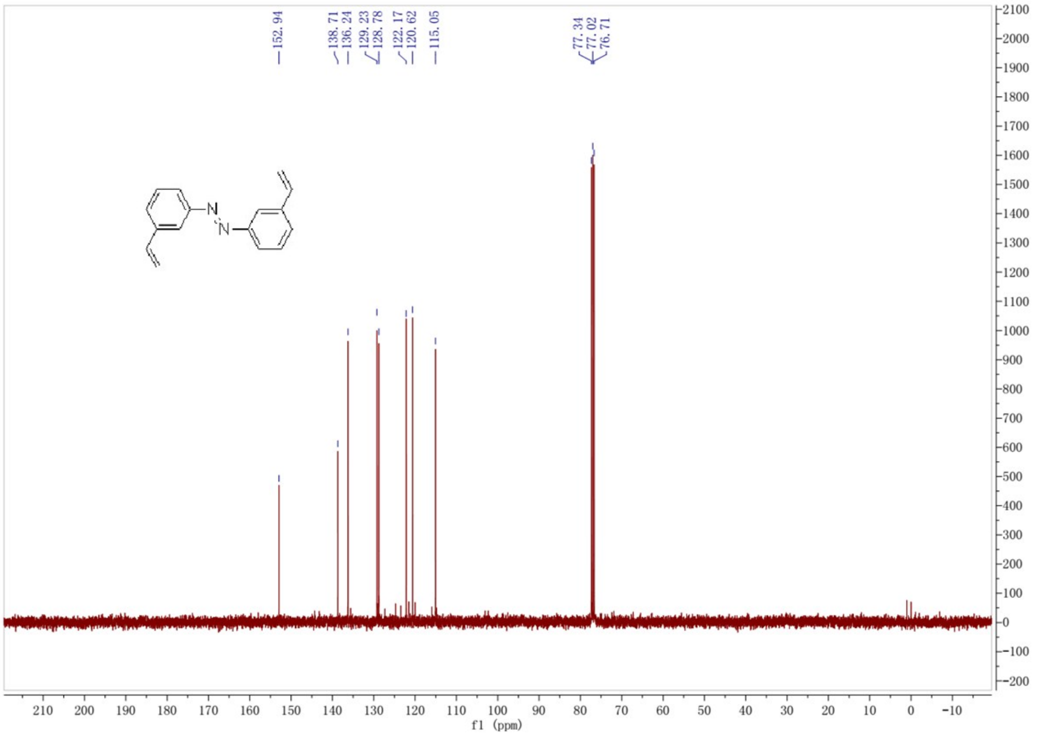
152.94
138.71
136.24
129.23
128.78
122.17
120.62
115.05
77.34
77.02
76.71
f1 (ppm)

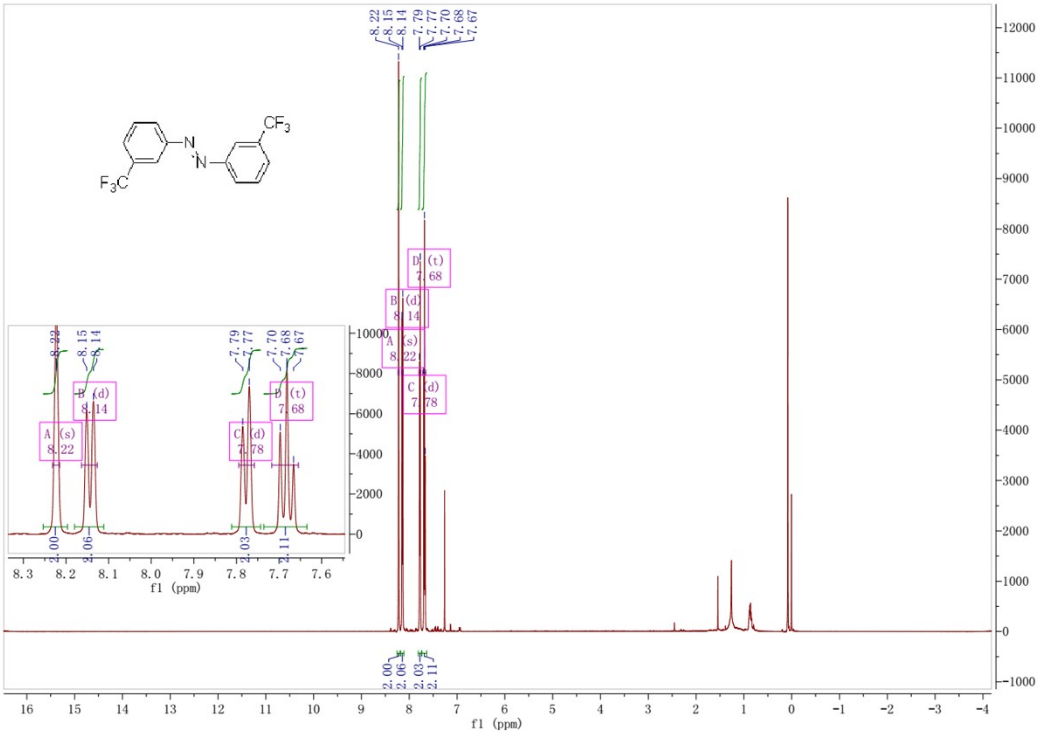
8.22
8.15
8.14
7.79
7.77
7.70
7.68
7.67
CF_3
F_3C
A (s) 8.22
B (d) 8.14
C (d) 7.78
D (t) 7.68
2.00
2.06
2.03
2.11
f1 (ppm)

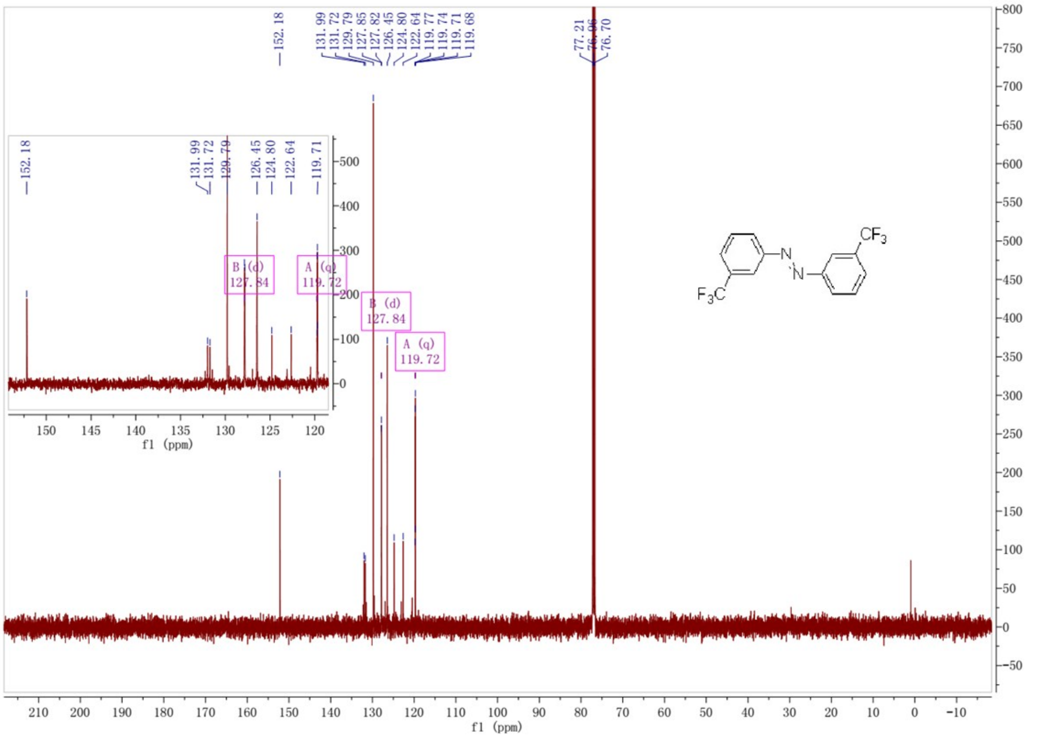

152.18
131.99
131.72
129.79
127.85
127.82
126.45
124.80
122.64
119.77
119.74
119.71
119.68
77.21
76.96
76.70
B (d)
127.84
A (q)
119.72
CF_3
F_3C
N
N
f1 (ppm)

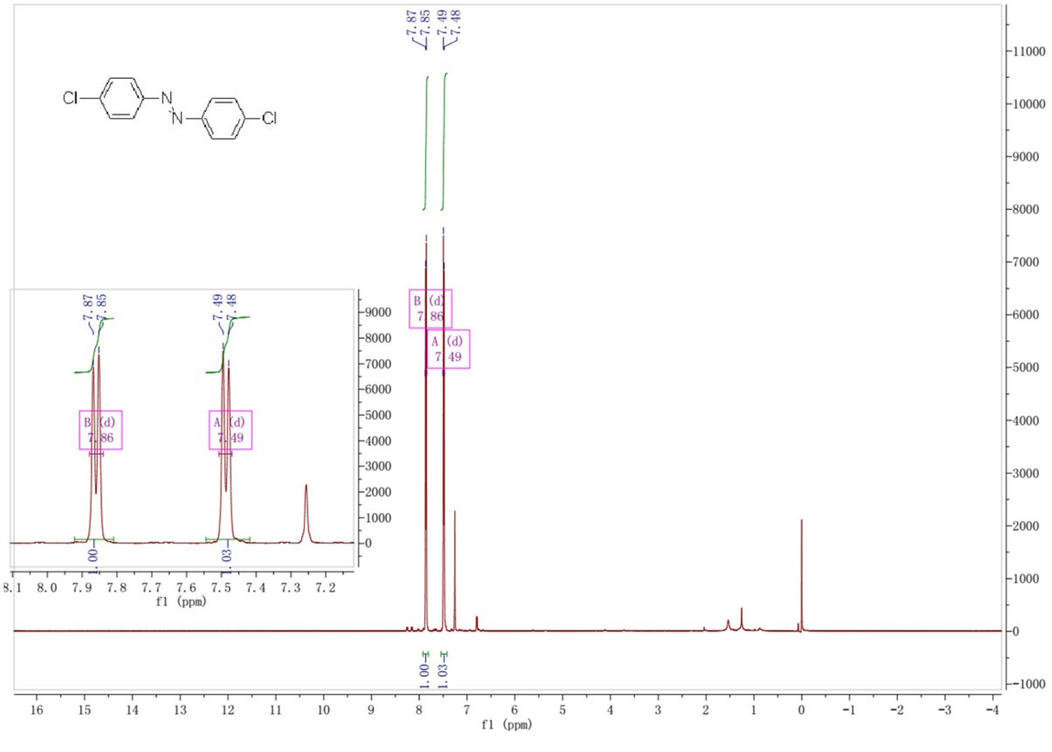

7.87
7.85
7.49
7.48
Cl
N
N
Cl
B (d)
7.86
A (d)
7.49
1.00
1.03
f1 (ppm)

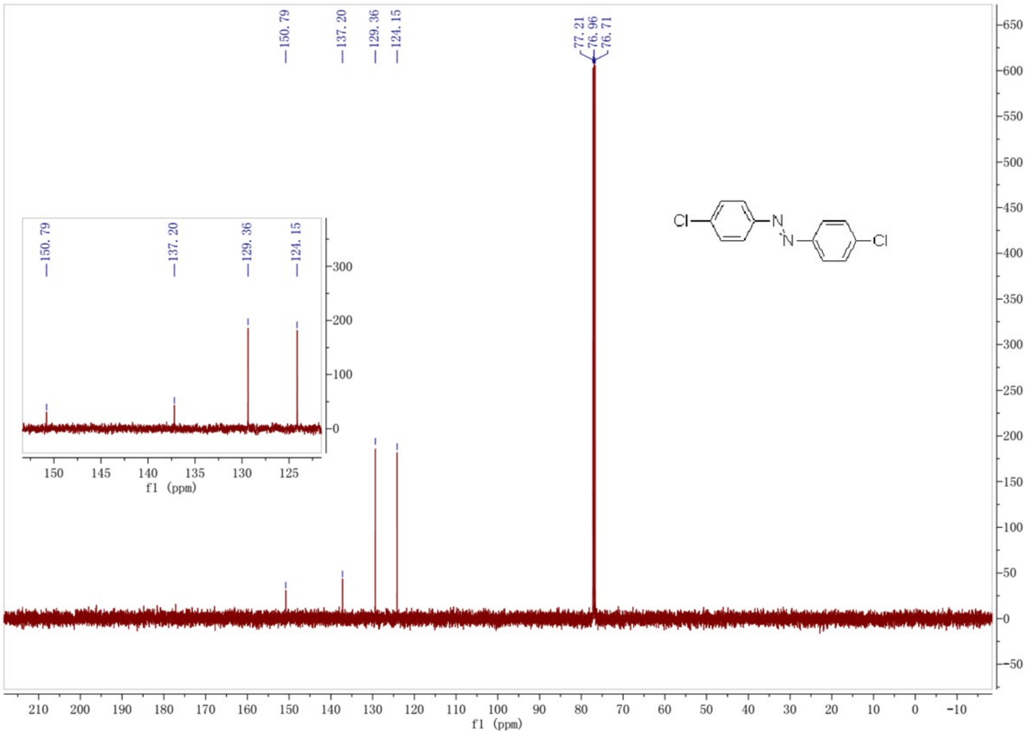

150.79
137.20
129.36
124.15
77.21
76.96
76.71
Cl
N
N
Cl
f1 (ppm)

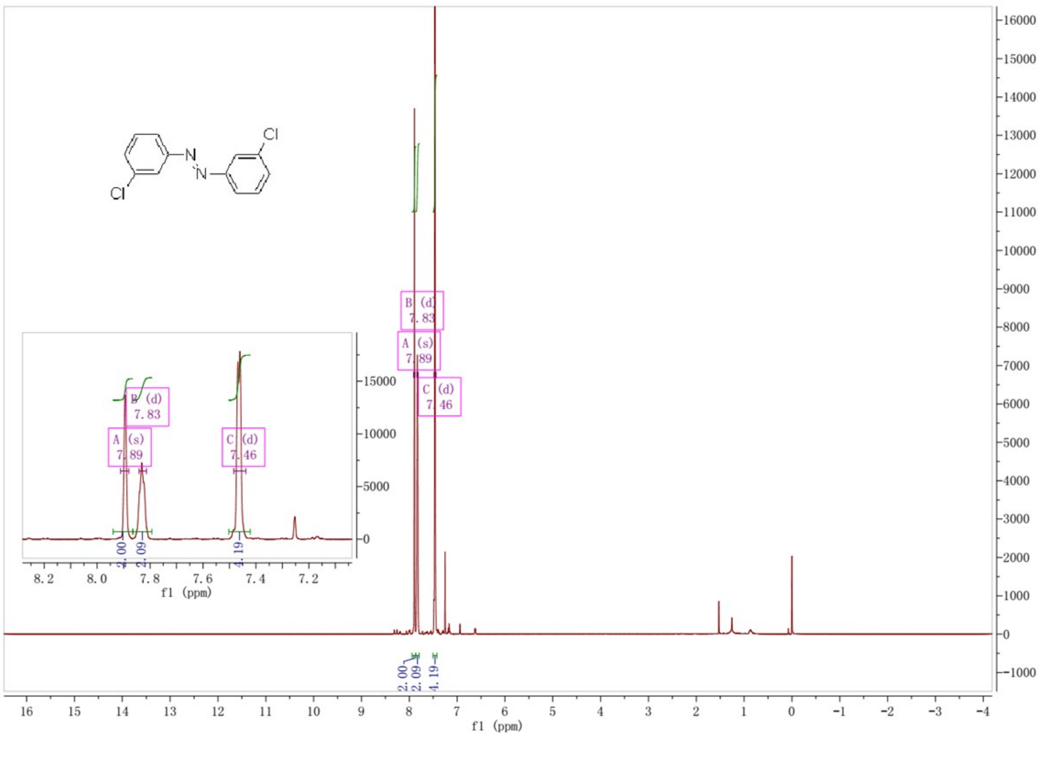

Cl
N
N
Cl
B (d)
7.83
A (s)
7.89
C (d)
7.46
2.00
2.09
4.19
f1 (ppm)

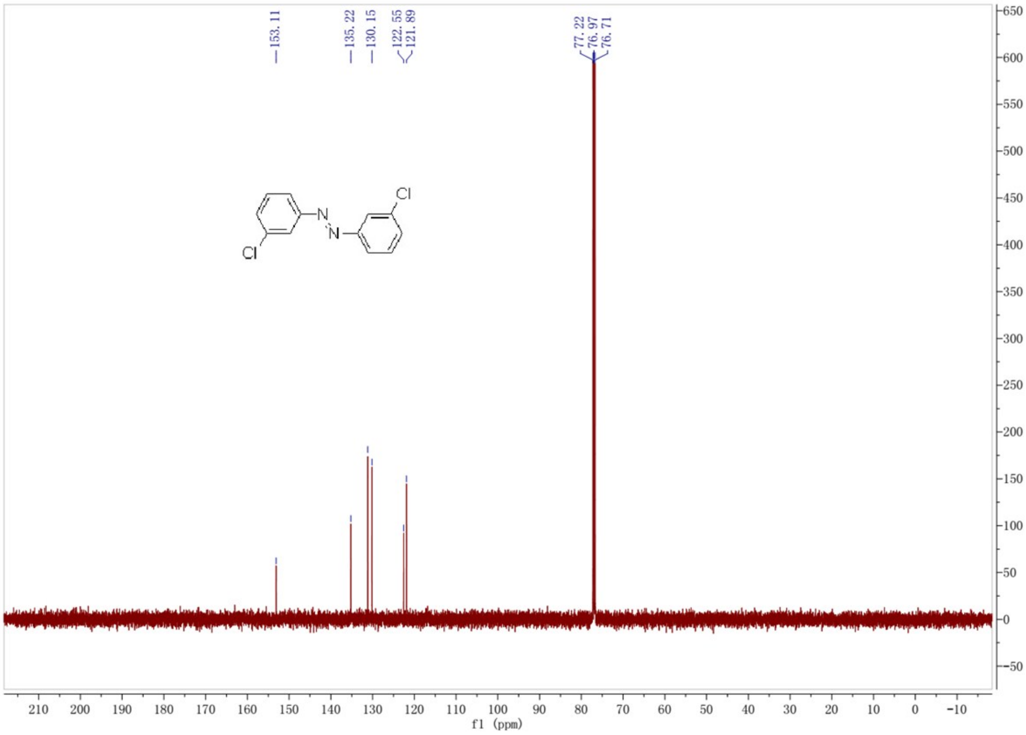

153.11
135.22
130.15
122.55
121.89
77.22
76.97
76.71
Cl
N
N
Cl
f1 (ppm)

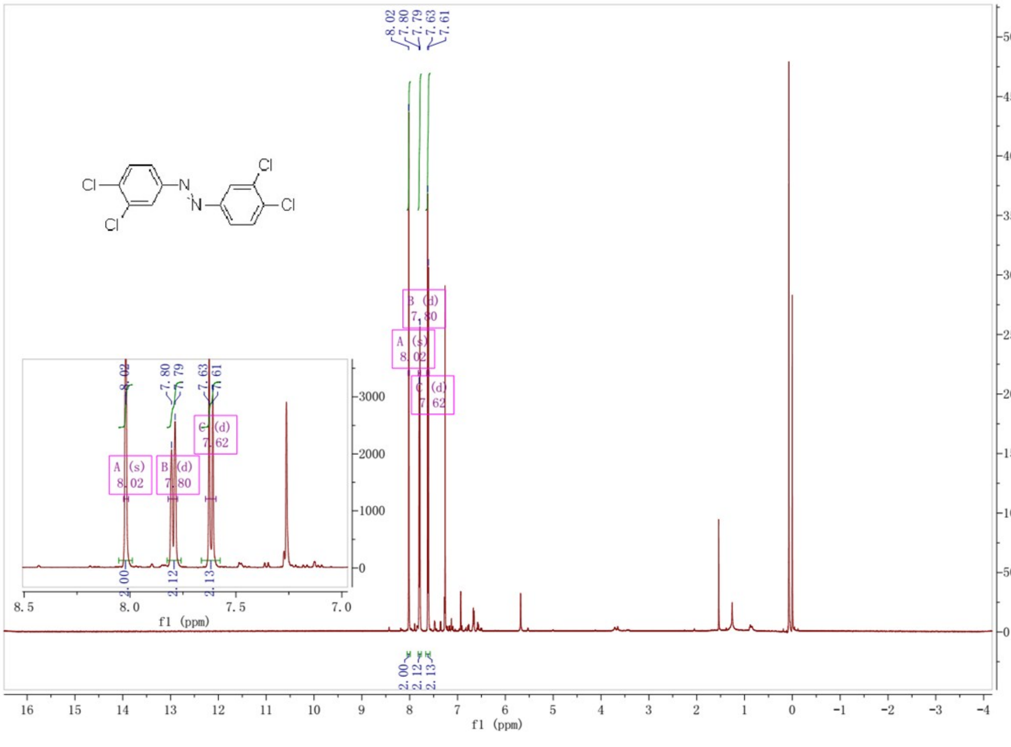

8.02
7.80
7.79
7.63
7.61
Cl
Cl
N
N
Cl
Cl
A (s)
8.02
B (d)
7.80
C (d)
7.62
2.00
2.12
2.13
f1 (ppm)

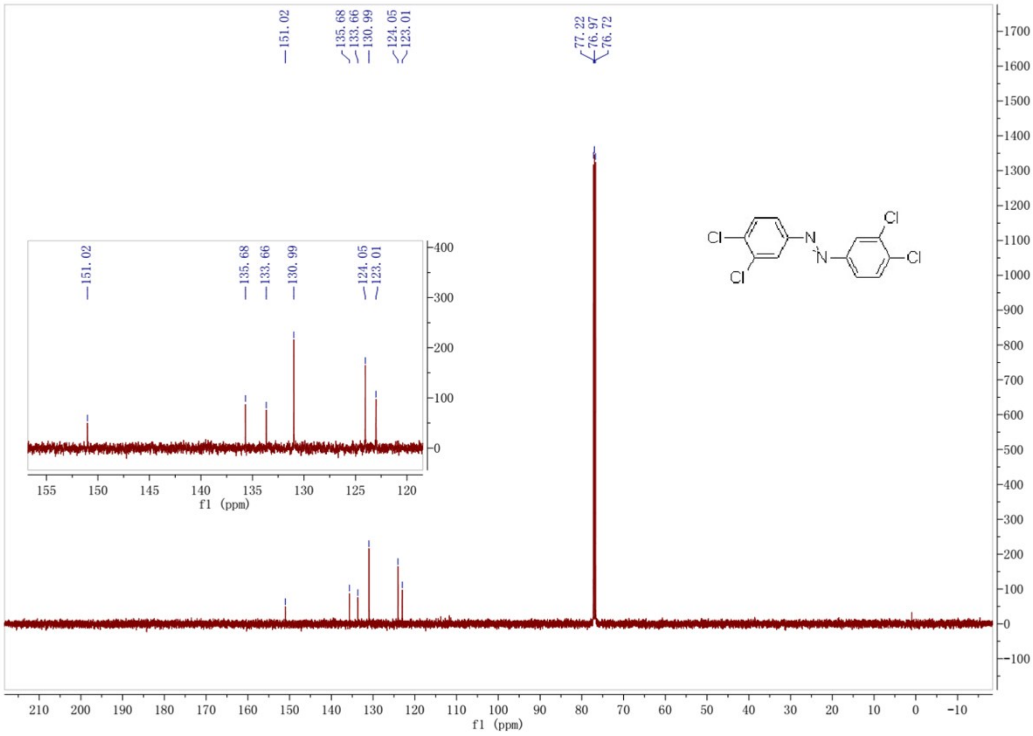
151.02
135.68
133.66
130.99
124.05
123.01
77.22
76.97
76.72
f1 (ppm)
Cl
N
N

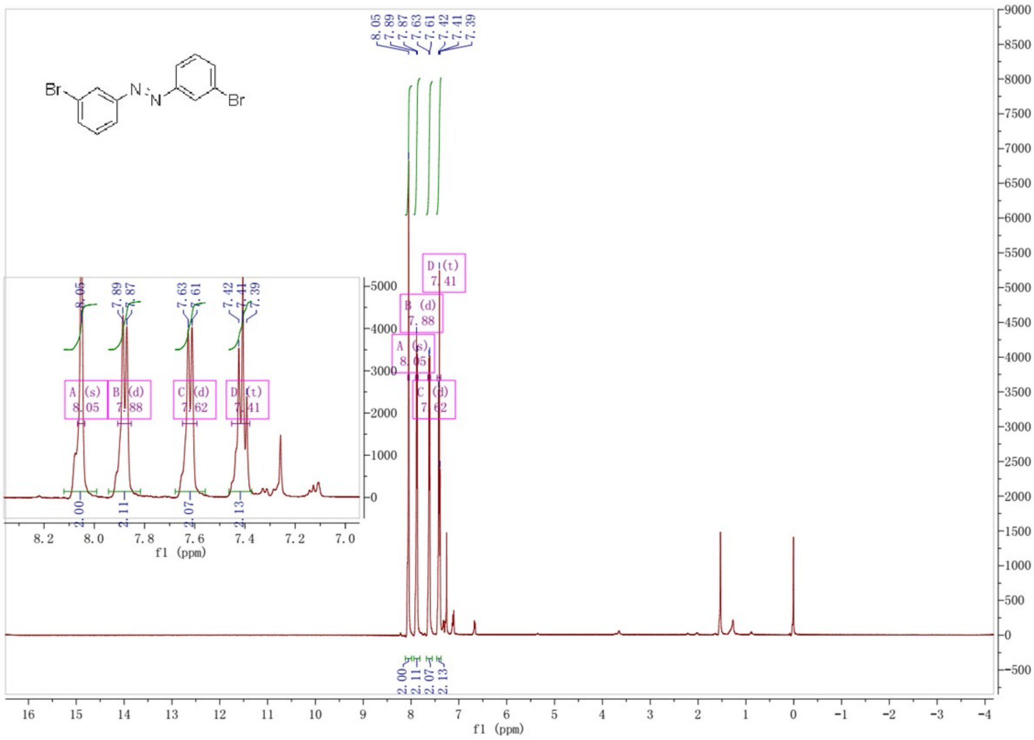
8.05
7.89
7.87
7.63
7.61
7.42
7.41
7.39
Br
N
N
A (s) 8.05
B (d) 7.88
C (d) 7.62
D (t) 7.41
2.00
2.11
2.07
2.13
f1 (ppm)

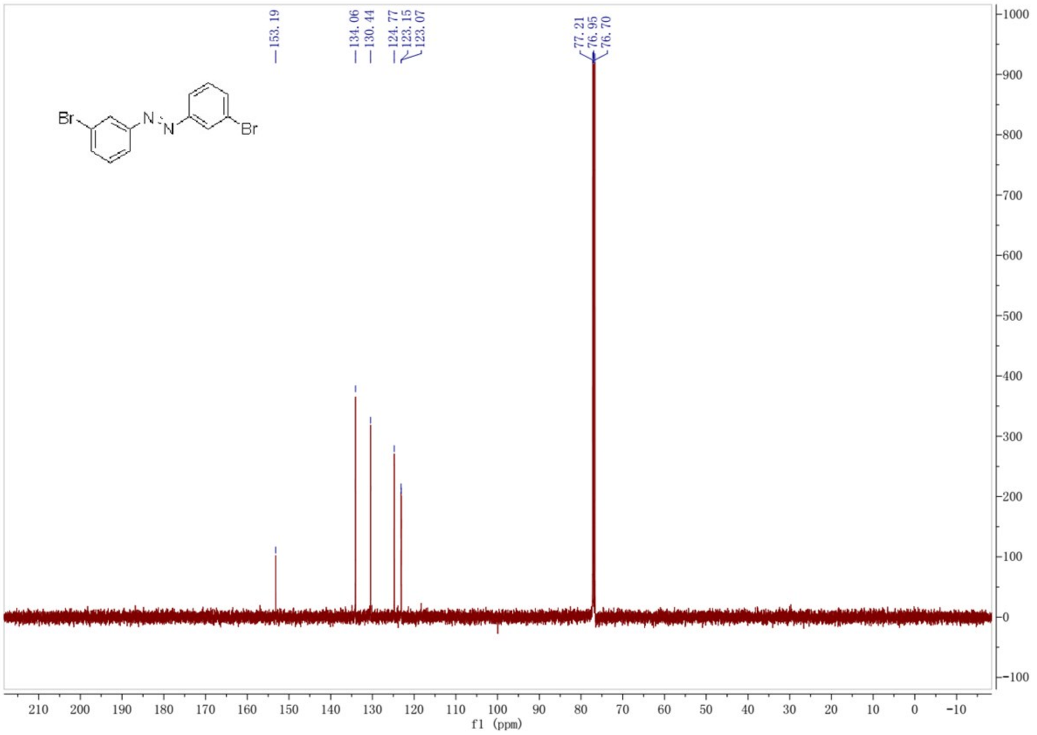

153.19
134.06
130.44
124.77
123.15
123.07
77.21
76.95
76.70
Br
N
N
Br
f1 (ppm)

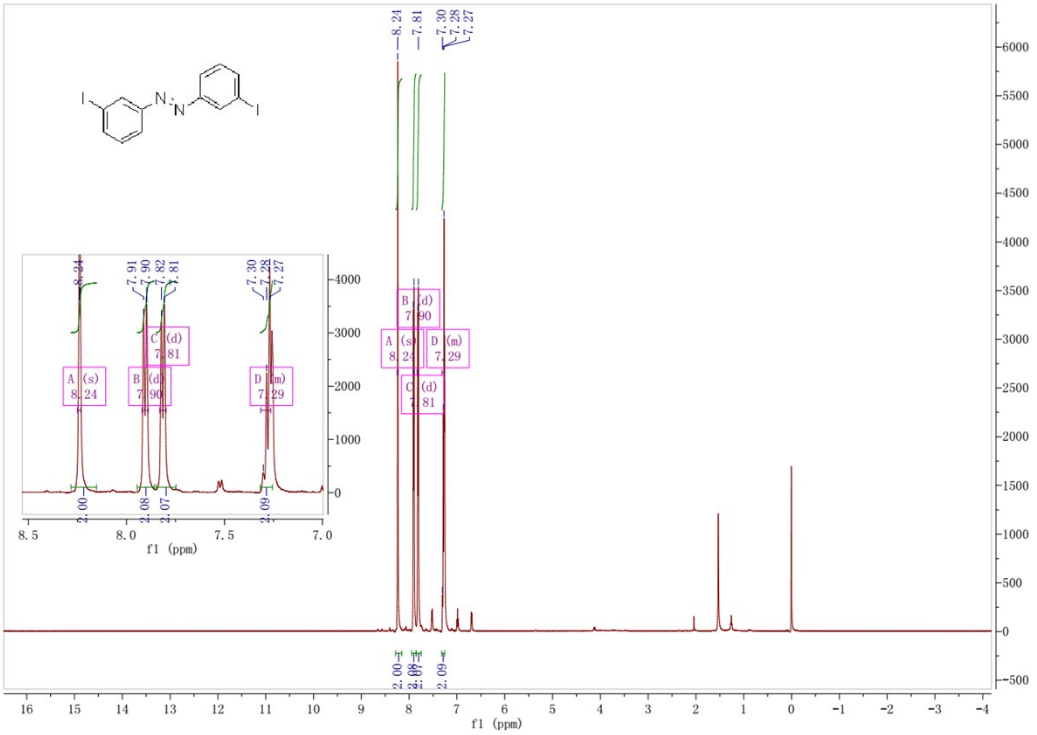

8.24
7.81
7.30
7.28
7.27
I
N
N
I
A (s)
8.24
B (d)
7.90
C (d)
7.81
D (m)
7.29
2.00
2.08
2.07
2.09
f1 (ppm)

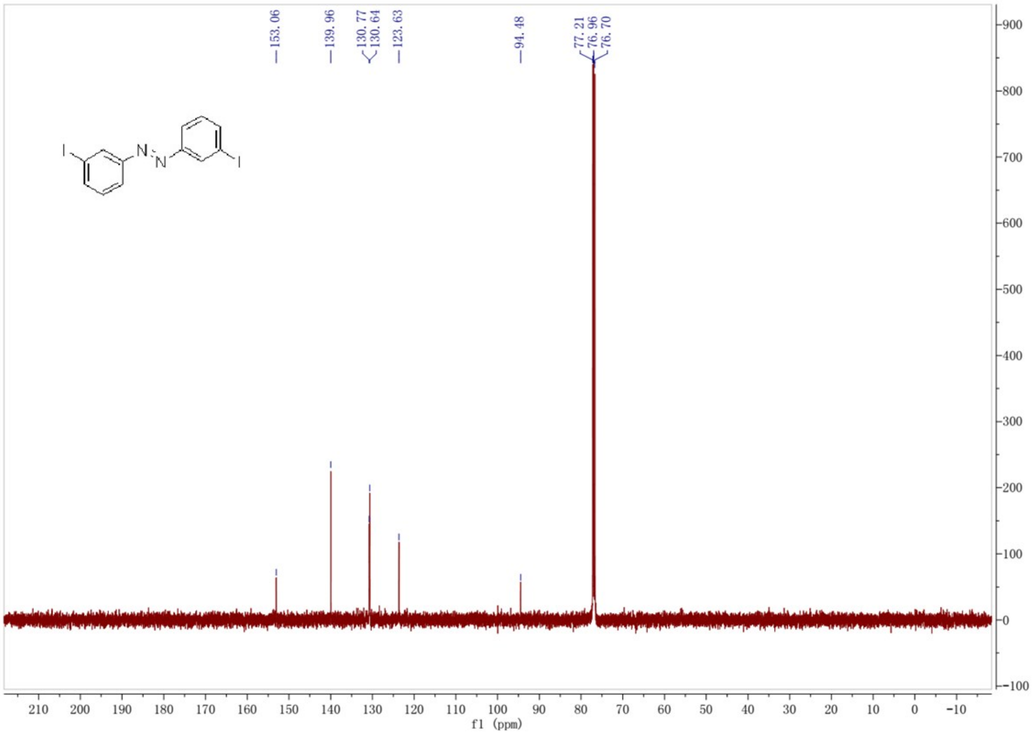

153.06
139.96
130.77
130.64
123.63
94.48
77.21
76.96
76.70
f1 (ppm)
